# Ancient Origins of Cytoskeletal Crosstalk: Spectraplakin-like Proteins Precede the Emergence of Cortical Microtubule Stabilization Complexes as Crosslinkers

**DOI:** 10.3390/ijms23105594

**Published:** 2022-05-17

**Authors:** Tina Paradžik, Iva I. Podgorski, Tanja Vojvoda Zeljko, Mladen Paradžik

**Affiliations:** 1 Laboratory for Biotechnology in Aquaculture, Division of Materials Chemistry, Ruđer Bošković Institute, 10000 Zagreb, Croatia; 2 Center of Excellence for Marine Bioprospecting (BioProCro), Ruđer Bošković Institute, 10000 Zagreb, Croatia; 3 Laboratory for Metabolism and Aging, Division of Molecular Medicine, Ruđer Bošković Institute, 10000 Zagreb, Croatia; iva.skrinjar@irb.hr; 4 Laboratory for Precipitation Processes, Division of Materials Chemistry, Ruđer Bošković Institute, 10000 Zagreb, Croatia; tanja.vojvoda@irb.hr; 5 Laboratory of Experimental Therapy, Division of Molecular Medicine, Ruđer Bošković Institute, 10000 Zagreb, Croatia

**Keywords:** cytoskeletal crosstalk, spectraplakin, cortical microtubule stabilization complex, focal adhesion, actin, microtubule, intermediate filaments, evolution, dystonin

## Abstract

Adhesion between cells and the extracellular matrix (ECM) is one of the prerequisites for multicellularity, motility, and tissue specialization. Focal adhesions (FAs) are defined as protein complexes that mediate signals from the ECM to major components of the cytoskeleton (microtubules, actin, and intermediate filaments), and their mutual communication determines a variety of cellular processes. In this study, human cytoskeletal crosstalk proteins were identified by comparing datasets with experimentally determined cytoskeletal proteins. The spectraplakin dystonin was the only protein found in all datasets. Other proteins (FAK, RAC1, septin 9, MISP, and ezrin) were detected at the intersections of FAs, microtubules, and actin cytoskeleton. Homology searches for human crosstalk proteins as queries were performed against a predefined dataset of proteomes. This analysis highlighted the importance of FA communication with the actin and microtubule cytoskeleton, as these crosstalk proteins exhibit the highest degree of evolutionary conservation. Finally, phylogenetic analyses elucidated the early evolutionary history of spectraplakins and cortical microtubule stabilization complexes (CMSCs) as model representatives of the human cytoskeletal crosstalk. While spectraplakins probably arose at the onset of opisthokont evolution, the crosstalk between FAs and microtubules is associated with the emergence of metazoans. The multiprotein complexes contributing to cytoskeletal crosstalk in animals gradually gained in complexity from the onset of metazoan evolution.

## 1. Introduction

Cells respond to various external stimuli (shear stress, strain, matrix stiffness, pressure, etc.) and can convert mechanical signals into biochemical signals, which is known as mechanotransduction. These force-induced signals are important for a variety of biological processes such as migration, differentiation, homeostasis, proliferation, and apoptosis [1].

External signals can be sensed by a variety of cellular receptors such as integrins, cadherins, selectins, and the immunoglobulin superfamily [2,3]. Among them, integrins are mainly responsible for communication by binding to extracellular matrix (ECM) proteins. Since their discovery to date, they have been the most intensively studied cell adhesion receptors and constitute the largest protein network that mediates and transmits signals from the ECM to the cytoskeleton [4]. Integrins are transmembrane molecules composed of α- and β- dimers, with the 18-α and 8-β subunits forming a total of 24 heterodimers. Instead of enzymatic activity, integrins possess the ability of bidirectional signalling, known as inside-out and outside-in signalling [5]. Signals are sensed by integrin heterodimers on the cell membrane and transduced in the cell via large complexes called focal adhesions (FAs). Immediately after integrin binding to ECM proteins, conformational changes within the integrin heterodimer enable signal transduction and activation of focal adhesion kinase (FAK), which facilitates further signal transduction through autophosphorylation of FAK and binding of SRC [6]. Integrin-mediated interactions between the ECM and the cytoskeleton play a critical role in cell movement, shape, and orientation, implying that cell adhesion processes are dynamic responses to external conditions such as mechanical strain [7]. Thus, there is increasing evidence of interactions between microtubules and actin filaments, and FAs appear to be a hotspot of cytoskeletal crosstalk [8].

The cytoskeleton of mammalian cells consists of three distinct cellular networks that differ primarily in size and protein composition: actin fibers or microfilaments (~7 nm), microtubules (~25 nm, MTs), and intermediate filaments (~10 nm, IFs) [9]. To function properly, all three parts of the cytoskeleton must be synchronized and work together. Each part of the cytoskeletal network can interact with other components of the cytoskeleton. This occurs through direct interaction via shared protein linkers that enable cytoskeletal crosstalk [10]. Therefore, proteins involved in crosstalk play a key role in the orchestrated regulation of the cytoskeletal response to mechanical/external signals and are responsible for various processes (migration, polarization, cell division, anticancer drug resistance), where the simultaneous action of the cytoskeleton can be viewed as an overall complex. Actin microfilaments and microtubules are found in all domains of life and support cellular movement and division in all Eucaryotes [11,12]. On the other hand, cytoplasmic IFs evolved in Metazoa from nuclear lamins, coinciding with the true origins of multicellularity and are considered an animal invention [13]. In the Chordata, the four major subtypes of IFs occurred sequentially, with extensive expansion of keratins associated with the transition from water to land [13].

Cell adhesion is widely considered to be a prerequisite for the evolution of multicellularity, including cell-cell adhesion and adhesion between cells and the ECM. Studies of distant animal relatives provide evidence for the early evolution of the animal-like cytoskeleton. Although unicellular, amoebozoans are characterized by a complex cytoskeleton [14]. *Entamoeba histolytica*, for example, has a cortical cytoskeleton and adhesion plates [15]. Additionally, recent analysis of the transcriptome of amoebae has revealed the presence of integrin genes [16]. Previous studies indicated early origins of integrin-mediated adhesion and signalling complexes that preceded the emergence of multicellularity in Opisthokonta (Fungi and Metazoa) [17]. In Amoebozoa, both integrin subunits were found mainly as a single paralog, whereas in unicellular opisthokonts, several different integrin subunits were discovered [16,17]. The genes encoding the major FA components that link these complexes to actin filaments, including vinculin, talin, and actinin, have been found in amoeba genomes [17]. In the sister lineage of Amoebozoa and opisthokonts, Apusomonads, the unicellular organism *Thecamonas trahens* possesses microtubules as well as bundles of microfibers that are located within the cell but are not connected to other cytoskeletal components [18]. Integrins have also been found in its genome [17]. *Capsaspora owczarzaki*, a unicellular opisthokont, can adhere to surfaces using actin-dependent filopodia. Integrin β2 and vinculin were found to be localized as distinct patches in these structures [19]. Another unicellular organism, *Corallochytrium limacisporum* has an actin cytoskeleton that shares features with both fungal and animal cells [20]. As an early-branching animal form, sponges (Porifera) have specific adhesion mechanisms. In addition, they have both types of adhesion systems found in other animals (adherent junctions and focal adhesions), arguing for an early origin of these structures in animal evolution [21]. In sponges, the presence of integrins that anchor the actin cytoskeleton through interactions with a large number of cytoplasmic proteins such as talin, vinculin, and paxillin has been studied [22]. In another early-branching animal, *Trichoplax adhaerens* (Placozoa), which possess only six distinct cell types [23], surface adhesion proteins (α/β-integrins, cadherins) were found together with its ECM-binding proteins (fibronectin, vitronectin). In addition, crosstalk proteins responsible for downstream signalling and actin filament organization (vinculin, paxilin, talin, and FAK) were also detected [24]. Furthermore, lamin-like genes have been identified as precursor genes of intermediate filaments in these animals [25].

While cytoskeletal crosstalk in humans has been analysed in several studies, to our knowledge, no study has combined the evolutionary aspects of cytoskeletal crosstalk between FAs, actin, MTs, and IFs. Thus, we summarized the proteins involved in the different aspects of the cytoskeleton. Proteins discovered at the intersections of different types of cytoskeletal filaments and FA complexes may be involved in crosstalk between individual filaments. Because little is known about the origin and evolution of cytoskeletal crosstalk in animals, we analysed its evolution in animals and their closest phylogenetic relatives using examples of protein-protein interactions that are known to be essential for cytoskeletal crosstalk in humans.

## 2. Results and Discussion

### 2.1. Retrieving of Cytoskeleton Crosstalk Proteins from Databases Pointed Dystonin as Major Regulator of Cytoskeletal Crosstalk

Over the decades, the human cytoskeletal system, which is composed of proteins that perform diverse and versatile functions, has been extensively studied. However, recent high-throughput studies of various complexes have revealed many additional proteins involved in cytoskeletal structure and function. Using the criteria described in Methods, we selected 1298 proteins involved in various aspects of cytoskeletal organisation. To get an overview of the relationships between the proteins of the cytoskeleton and their grouping in general, we clustered all proteins of the cytoskeleton based on sequence similarities using the tool CLANS in 3D space (Supplementary Figure S1). Using the “find cluster” in-tool option, we obtained 12 groups of >10 proteins. By far the largest group of structural proteins is the intermediate filament cluster (IF), which has the largest divergence in vertebrates [13]. As an indication of their young evolutionary age, almost all intermediate proteins fell into this one cluster, whereas proteins belonging to other types of filaments are more randomly distributed (Supplementary Figure S1) and form only isolated clusters. Another large CLANS cluster consists of various types of kinases (mainly serine/threonine and tyrosine). This is not surprising, considering their importance in the rapid recruitment of proteins involved in signal transduction processes. A total of 77 kinases have been identified in processes related to cytoskeleton remodelling (about 15% of the kinases in the human kinome) [26].

We were particularly interested in uncovering the proteins at the intersections between datasets containing proteins of the cytoskeleton and focal adhesions (Supplementary Table S1, Sheets 1–4).

**Figure 1:**
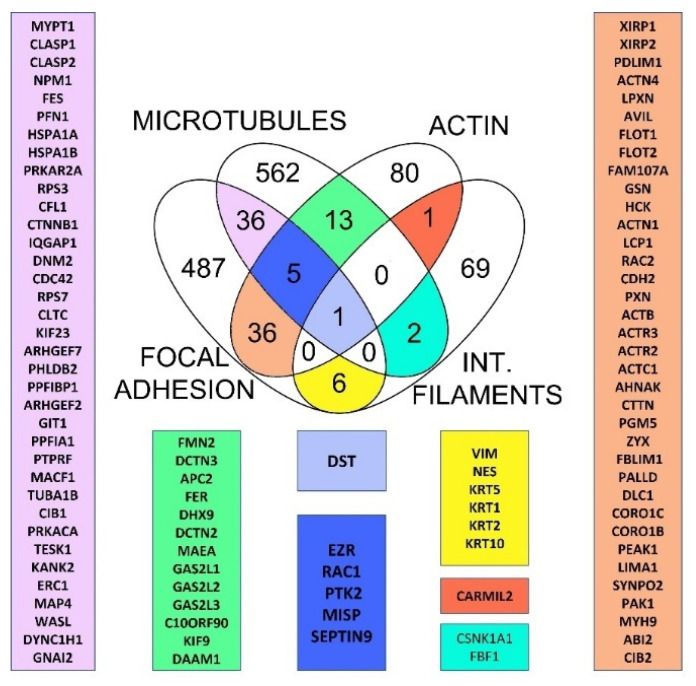
Putative cytoskeletal crosstalk proteins in humans. Venn diagram depicting 100 common proteins when using different datasets related to microtubules (MT), focal adhesions (FA), intermediate filaments (IF), and actin filaments. Dystonin (purple) was found in all datasets, whereas crosstalk proteins found in the MT, FA, and actin datasets are shown in a dark blue rectangle. Proteins at the crossing points between MT and FA (pink), MT and actin (green), FA and actin (beige), IF and MT (light blue), IF and actin (red), IF and FA (yellow) were generated as described in the Section 4.

The overlapping of the retrieved datasets displayed only one common protein, dystonin (DST, also known as BPAG1 or BP230) (Figure 1). Together with a microtubule-associated crosslinking factor 1 (MACF1) DST belongs to the spectraplakin family of proteins, a family of cytoskeletal regulators capable of binding different types of cytoskeletal filaments [27,28]. This is achieved through different binding modules present in these proteins. The calponin homology (CH) domain is involved in actin binding, while GAS2 (growth arrest specific 2)-related domain interacts with microtubules (also known as GAR domain) [29]. In addition, spectraplakin proteins possess a plakin domain that contains up to nine spectrin repeats (SR1-SR9) and a Src-homology 3 (SH3) domain in the middle [30]. Finally, the plectin (or plakin) repeat domain (PR) in spectraplakin and plakin proteins serves to bind IF proteins [31,32].

DST is important for maintaining the integrity of FAs and plays a key role in cell-substrate adhesion and cell spreading [33]. Moreover, it has been found to localize with hemidesmosomes in a region where keratin filaments are associated with hemidesmosomal plaque [34]. Additionally, DST interacts directly with MTs and their plus-end proteins EB1 and EB3 [28,35].

**Figure 2:**
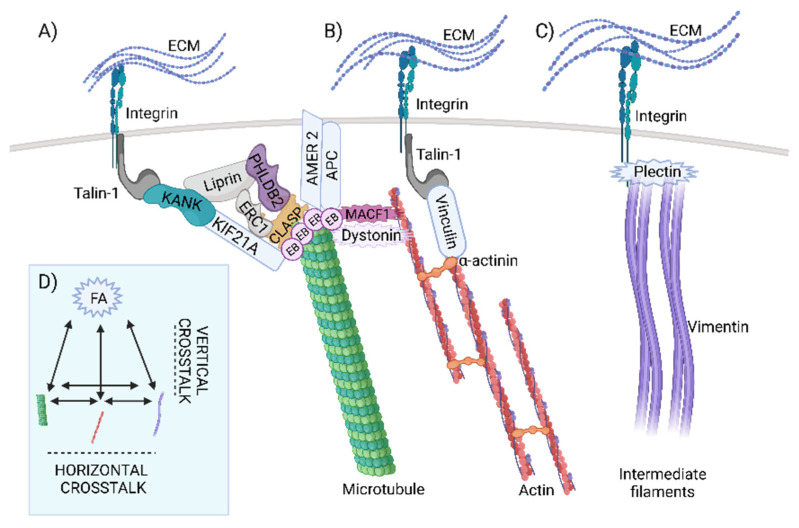
Schematic representation of vertical and horizontal crosstalk. (**A**) After binding to ECM proteins, the signal is transduced to focal adhesions via integrins. Proteins of the cortical microtubule stabilization complex (liprins, ERC1, PHLDB2, CLASPs, AMER2, and APC) play an important role in force transmission to microtubules, while direct binding of KANK to talin-1 and KIF21A contributes to microtubule recruitment to the outer rim of focal adhesions. (**B**) Force transmission to actin filaments is facilitated by the binding of talin-1 to cytoplasmic integrin tails that recruit mediators such as vinculin and actinin alpha and help to transduce the signal to actin filaments. (**C**) ECM-integrin-mediated force transmission to intermediate filaments is facilitated by binding of the β4-subunit and plectin, whereas depletion of vimentin blocks β4-enhanced invasion. In addition, binding of plectin to the β4 subunit is responsible for recruitment to focal adhesions and cell spreading. (**D**) Simplified scheme of vertical and horizontal crosstalk.

Five proteins were found in the intersection of the datasets of MT, FA, and actin: septin 9, MISP, ezrin, RAC1, and FAK. While RAC1 and FAK are well-studied effector proteins involved in various pathways related to activation of FAs and cytoskeletal proteins [36,37], other proteins emerged at the intersections during analysis. Septin 9 colocalises and associates with MTs, whereas its depletion leads to thinning of the MT network [38]. Moreover, septin 9 increases the length of MT, by suppressing MT catastrophe [39]. Additionally, MISP has been shown to interact directly with ezrin, while activated ezrin regulates MISP levels by competitively binding to actin binding sites on the cell cortex. In this way, the prevention of excessive MISP levels in the cell cortex is regulated, allowing proper polarization and optimal positioning of the mitotic spindle [40]. According to data retrieved from available databases, signal transduction through FAs to MTs and actin filaments is mediated by at least 36 different proteins, whereas to IF it is mediated by six proteins. Simplistically, signal transduction from FAs to different filament types can be considered “vertical”, whereas signal transduction between filaments can be considered “horizontal” crosstalk (Figure 2).

Two parameters contribute to the total number of proteins relevant to crosstalk: (1) the number of relevant studies performed to date together with the number of database entries for these studies, and (2) the total number of existing connections. First, we found that some proteins that have already been characterized in detail with respect to the crosslinking of different components of the cytoskeleton are not present in certain databases and this could be considered as one of the main limitations using this approach. A perfect example is the plakin protein plectin, which is known to crosslink FA, actin, and IF [41,42,43,44], but is not yet present in actin and IF databases. Second, we can discuss the number of connections that exist in terms of evolutionary age. Since IFs are by far the youngest cytoskeletal structures [11], there was not enough time to evolve as many connections as there are to MT and actin filaments. The same logic can be applied to “horizontal” links between different filament types. We revealed 13 overlapping proteins between actin filaments and MT and only 2 or 1 at the intersection of MT or actin datasets to IFs, respectively (Figure 1). The role of IFs in signal transduction and mechanotransduction remains poorly understood [45], and identification of new crosslinking proteins is expected in the future. In any case, the number of proteins intersecting FAs and both MTs and actin is by far the largest compared with other intersections. This is consistent with the important role of FAs in cytoskeletal control.

In summary, FAs communicate with the cytoskeleton through many different connections in a “vertical” manner. In contrast, fewer players have been identified for “horizontal” crosstalk. Among these proteins, spectraplakins are by far the best characterized and studied. In addition, they may also act as “vertical” linkers by reaching out to FA. Dystonin is pointed as the only protein that connects all elements of the cytoskeleton and FA, whereas several other proteins have been found to be involved in the “triple” crosstalk. In the second part of this study, we focus on the evolution of the multiple crosslinkers, with particular attention to the spectraplakins and the “vertical” links. These analyses may help us understand how and when specific hotspots of cytoskeletal crosstalk evolved.

### 2.2. Evolutionary Conservation of Cytoskeletal Crosstalk Proteins

**Figure 3:**
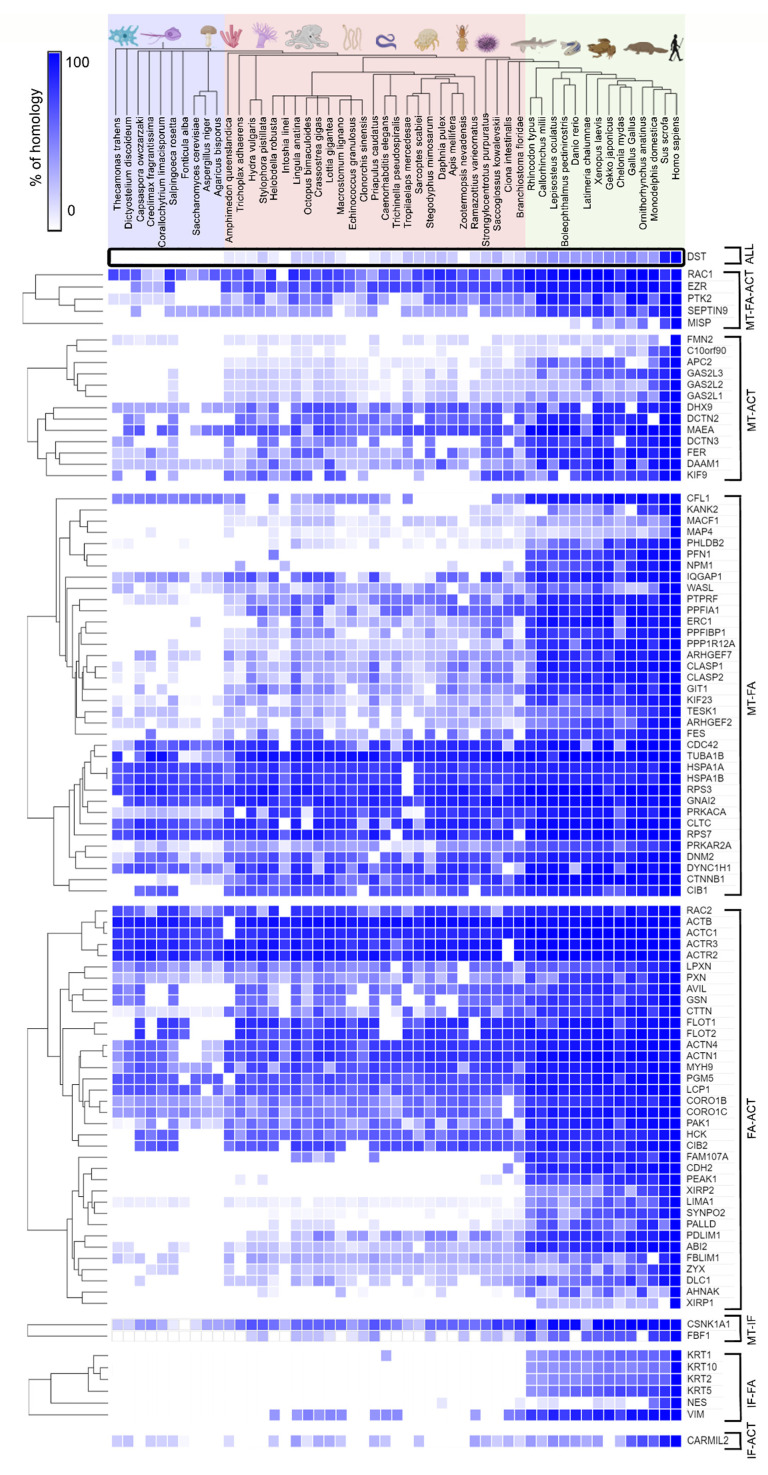
Evolutionary conservation of cytoskeletal crosstalk proteins. Heatmap representing percentage of homology for crosstalk proteins found at the crossing points of the microtubules (MT), focal adhesions (FAs), intermediate filaments (IFs), and actin (ACT) in human cytoskeleton datasets. The proteins found at the crossing points were classified according to their occurrence in the respective datasets, and hierarchical clustering (Euclidean distance algorithm and average linkage method) was performed for each group separately using the Morpheus online tool.

To obtain a general overview on the evolution of the obtained crosstalk proteins, we searched for the closest homologs in our organism dataset using human crosstalk proteins as queries with the BLAST2GO tool. The results of this analysis revealed dynamic evolutionary patterns within different cytoskeletal intersections (Figure 3). As expected, most vertebrate proteins (from *Rhinocodon typus* to *Homo sapiens*, green rectangle) show a high percentage of similarity to human homologs, while many corresponding homologs show lower similarity to proteins from amoebae, unicellular opisthokonts, and fungi (organisms in the purple rectangle, from *T. trahens* to *Agaricus bisporus*).

Actin and tubulin are considered to be present in the common ancestor of all life [11], while the components of FA are found in the Amorphea clade, with Amoebozoa and Apusozoa as the most distant animal relatives [17] (Supplementary Figure S2). Interestingly, homologs of most proteins found at the intersection of actin and microtubules are missing in more distant organisms, such as unicellular opisthokonts and early-branching Metazoa. Contrary, intersections of MT or actin with evolutionary younger FAs show high conservation in half of the homologs in all organisms from our dataset (Figure 3). This may reflect the important role of FAs in orchestrating cytoskeletal crosstalk that predates animal origins. Finally, IFs are by far the youngest types of filaments that evolved from nuclear lamins and are closely associated with animals divergence [46]. Few proteins are found at intersections with IFs, and their overall homology with proteins of organisms other than Vertebrata is low. However, the comparison of full-length proteins does not necessarily reflect the function of the potential homolog in simpler organisms. It is likely that the conservation of protein interaction domains does not exceed the significance threshold established in the previous analysis because of the small size, despite the potential conservation of interaction domains. Although high sequence homology is indicative of protein function, analysis of specific domains and motifs involved in protein-protein interactions, in combination with phylogenetic analysis, may provide more accurate answers to the evolution of cytoskeletal crosstalk. The evolution of some crosstalk proteins and their domains that are important for cytoskeletal filament binding, and whose role has been experimentally confirmed in humans, is therefore reviewed and analysed in the following chapters.

### 2.3. Evolution of Horizontal Crosstalk Interaction Hot Spots

**Figure 4:**
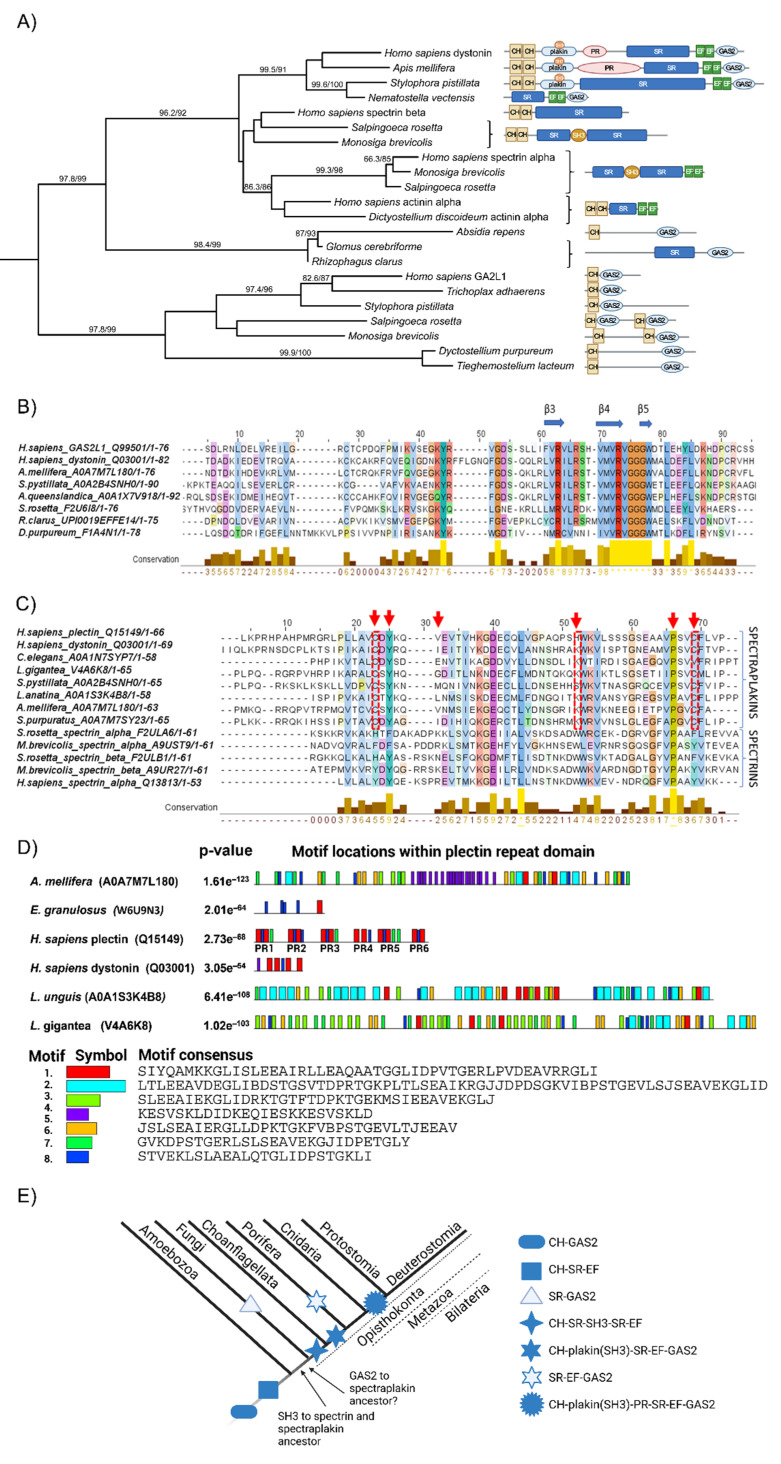
Analysis of spectraplakin proteins and domains in animals, and its closest relatives. (**A**) Phylogenetic relationship of different spectrin, spectraplakin, and GAS2 proteins. The phylogenetic tree was reconstructed based on concatenated CH, GAS2, SR1 and SR4 (or the last SR in spectrins, spectraplakins and SR-GAS fungal proteins) protein domains using a maximum likelihood method. The numbers indicate SH-aLRT and UFBoot values (only >80 values are shown). Tree is midpoint rooted. Schematic domain representation of corresponding proteins is designated on the right: CH —calponin homology domain, PR—plectin repeat, SR—spectrin repeat, GAS2—growth arrest specific 2 (Gas2)-related domain, SH3—Src-homology 3 domain. (**B**) GAS domain conservation. GAS domains from spectraplakins and GAS proteins belonging to phylogenetically distant organisms were extracted using InterProScan and aligned. β plates 3, 4, and 5 indicate MT interacting site. (**C**) SH3 (Src-homology 3) motif conservation in spectrin and spectraplakin proteins. Alignment of SH3 motifs from spectraplakin and spectrin proteins were identified by InterProScan. Putative interacting sites are designated with red arrows. Red boxes indicate change from spectrin aromatic residues to smaller side-chain amino acids. Overall conservation is indicated below alignment, (**B**,**C**) 0→* increasing amino acid (aa) residues conservation, * = aa residues in all sequences are conserved. (**D**) Plectin repeat domain (PR) was identified by InterProScan in human spectraplakins/plakins and different Protostomian spectraplakins. Identification of conserved repeated motifs was obtained by MEME suite. (**E**) Proposed evolutionary scenario of spectraplakins and their domains and other related proteins.

To understand the evolutionary relationships of the studied crosstalk proteins and to track their abilities to interact with different components of the cytoskeleton, we have analysed their orthologs in different Opisthokonta groups, as well as Amoebozoa as an outgroup. Intersection analysis revealed dystonin, a giant protein that belongs to the spectraplakin family, as the only protein found in all datasets, as mentioned earlier (Figure 1). Clustering of all cytoskeletal proteins found in the database search grouped all spectraplakins and plakins into a single cluster (Supplementary Figure S1, “spectrin” cluster, Supplementary Table S1, Sheet 5). In addition, non-spectrin proteins such as filamin and plastin are also part of this cluster, probably due to the presence of the calponin homology (CH) domain. Using phylogenetic analyses and analyses of conserved domains, we elucidated the early evolutionary history of spectraplakins (Figure 4).

The members of spectraplakin family are known to be important players in cytoskeletal crosstalk. As described in Section 2.1 human spectraplakins are multidomain proteins that comprise several interacting modules. The CH domain is normally located at the N-terminal end of the protein and consists of two tandem subdomains, CH1 and CH2. This domain is widely distributed in the different kingdoms of life. In Amorphea, actinin alpha is the simplest member of the group harbouring both CH domains, whereas giant and multidomain spectraplakins are advanced integrators of the cytoskeleton. Actinin alpha is considered the ancestral form of spectrin-based proteins [47,48]. Actinin itself probably evolved from the protein-containing CH domain and a spectrin-like repeat [49].

The spectrin repeat (SR) is the second feature of spectraplakins and consists of approximately 100–115 amino acids that can form three α-helices. The spectrin motif is also an ancient evolutionary invention [50], and we detected it outside of Amorphea; however, it is usually present as a single domain outside of this clade. Ancestral actinin probably had two spectrin repeats, corresponding to SR1 and SR4 in modern actinin alpha [51]. Spectrin proteins evolved from an actinin ancestor through multiple intragenic duplications of spectrin repeats and serve as crosslinking molecules between actin filaments and integral membrane proteins [52]. Previous studies have shown that SR1 of actinin corresponds to SR1 in spectrin beta, whereas the last SR in spectrin alpha corresponds to SR4 of actinin, indicating a split in ancestral spectrin [48]. We were unable to discover spectrin homologs outside the Opisthokonta group using BLAST or SMART, HMMER, or InterPro-conserved domain search. However, we have found highly conserved spectrin homologs in the plant *Rhodamnia argentea* that were also experimentally characterized recently. Nevertheless, the authors had no explanation for the presence of this protein in the single plant species [53]. Within the unicellular opisthokonts, both spectrin types, alpha and beta were previously identified in the Choanoflagellata *Monosiga brevicolis* [52]. In addition, we have found both spectrins in another Choanoflagellata, *Salpingoeca rosetta*. Phylogenetic analysis placed all analysed spectrins in a strongly supported clade (Figure 4A), together with actinins alpha, confirming previous studies on the phylogenetic relationships of these two proteins.

In the C-terminal region of spectraplakins, two autonomous domains can be identified, EF hand (EF1-EF2) and GAS2. GAS2 alone is sufficient for microtubule binding and likely enhances EF1-EF2-MT engagement [54]. While the EF hand is found in many Ca^2+^-binding proteins throughout the kingdom of life [55], the GAS2 domain is more common in unicellular opisthokonts, Fungi, and Amoebozoa. However, we also found this domain outside the Amorphea clade, namely in the Alveolata species *Stylonychia lemnae*. In the phylogenetically distant Amorphea, such as Amoebozoa, Fungi or Choanoflagellata (Figure 4A), the GAS2 domain is usually found in combination with the CH domain (Figure 4A), similar to the human G2L1 and G2L2 proteins [29], and it probably has the same function in connecting actin filaments to microtubules. The conserved region spanning the β3-β5 sheets is involved in GAS2-microtubule binding [56]. This region is highly conserved in diverse proteins from species as distant as unicellular premetazoans and slime molds (Amoebozoa), indicating a conserved function in MT binding (Figure 4B).

In addition, both plakin and spectraplakin proteins possess a plakin domain that characteristically contains up to nine spectrin repeats (SR1-SR9) and a Src-homology 3 (SH3) domain in the middle [30]. Spectraplakin SH3 domain likely has common origins with the SH3 domain from the spectrins (Figure 4C). Interestingly, in Choanoflagellata (*M. brevicolis* and *S.rosetta*), the SH3 domain can be detected in both spectrin alpha and beta (Figure 4A,C). Contrary, the SH3 motif is present only in alpha version of human spectrin [57]. In spectrins beta of early-branching Metazoa (Porifera, Cnidaria, and Placozoa), we did not identify a SH3 domain, indicating a loss of this domain from a spectrin beta ancestor during early animal evolution. The SH3 domain is commonly known to mediate protein-protein interactions [58,59]. Recent work describes this domain as a mechanosensor that plays a critical role in mechanotransduction at hemidesmosomes [58,59]. Spectrins bind to proline-rich domains of other proteins via the SH3 motif [60]. The plakin domain is also known to interact with various proteins [61,62,63]. However, it appears that the affinity of this domain for proline-rich motifs is different [64]. In the spectraplakins, several important aromatic residues are replaced by amino acids with smaller side chains. We analysed how this motif is conserved in the plakin domains of protostomes and deuterostomes and compared it to spectrin SH3 from unicellular opisthokonts (Figure 4C). It appears that the origins of spectraplakins involve specific modifications of this SH3 motif from primordial spectrin that are conserved from cnidarian to human spectraplakins.

In preliminary phylogenetic analysis homologs from Porifera and Placozoa (*A. queenslandica:* A0A1X7V918 and *T. adhaerens:* B3S1H0), grouped with Eumetazoa spectraplakins (not shown). However, they do not have all spectraplakin modules present in higher animals, missing CH plakin domains and a large proportion of spectrin repeats. Considering the similar domains arrangement in the spectrins (CH-SR-EF), and the presence of the SH3 domain in both spectrins and spectraplakins, we can speculate that these proteins are shortened versions of an ancestral protein-containing CH-SR-EF-GAS2 domain organisation. A protein that can be considered true spectraplakin is found within *Stylophora pystillata* (Cnidaria) proteome. This protein has plakin domain inserted between tandem spectrin repeats, along with the CH domain at N-terminus and the GAS2 domain located at the C terminus of the protein. Contrary, another Cnidaria species, *Nematostella vectensis*, possess another “truncated” version of spectraplakin (Figure 4A).

The last common feature of spectraplakins, the plectin repeat domain (PR), is found in the Deuterostomia spectraplakins and the plakins, and Protostomia spectraplakin homologs (Figure 4A,D). The PR domain represents a new interaction hotspot in the evolution of Metazoa as a binding site for the animal-specific coil-1 region of IF proteins [31,32]. The single PR module is composed of 38 aminoacids tandemly repeated five times. Different PR modules are connected by less-conserved linker sequences of variable lengths [65] and form PR domains (Figure 4A). These modules are variously combined in different human spectraplakins and plakins and their isoforms [32] and serve to bind to different types of IF proteins. For example, human plectin has six PRs arranged in a specific manner. In addition to PRs, the linker region downstream of PR5 is likely involved in the interaction with IFs [66,67,68]. Mechanistically, the basic grooves of PRs and linkers fit into the coiled-coil rods of IFs. Using various domain search tools, we were unable to detect this domain outside of Eumetazoa. Within the Protostomia proteomes in our organism dataset, we found PRs in the spectraplakins of Mollusca, Brachiopoda, Insecta, and Platyhelminthes. As shown in Figure 4D, PRs can be identified by InterProScan but the architecture of the PR domains significantly differs between analysed spectraplakin proteins, as shown by MEME analysis, with PRs showing divergence in terms of number of repeated modules. It seems that Protostomia spectraplakins have a large number of PR modules compared to human dystonin (spectraplakin) and plectin (plakin) (Figure 4D). The overall sequence homology with human PR is also low, about 20%. Interestingly, arthropods are known to lack cytoplasmic IF proteins [69]. We found that *Apis mellifera* (honey bee) is one of the Protostomia species that have PRs in their spectraplakin proteins. Considering convergent IF evolution [13], it can be assumed that the PR regions in the spectraplakin proteins of protostomes and especially insects have evolved to perform different functions or bind to different interaction partners than in the spectraplakin and plakin proteins of Vertebrata. Moreover, the divergence of the interacting PR sites of the vertebrate spectraplakin and plakin proteins is likely associated with expansion of IFs within this group of organisms.

Finally, to elucidate phylogenetic relationships and ancestry of Vertebrata multidomain spectraplakins and plakins, we analysed three spectraplakin and plakin family proteins in humans (Supplementary Figure S3). Phylogenetic analyses show that the human-like plakin is derived from the common ancestor of Tunicata and Vertebrata, probably from a common spectraplakin ancestor. Duplication to human-like dystonin and MACF1 occurred later, before the divergence of the Vertebrata. These proteins can effectively link different cytoskeletal elements in a time- and cell-type- or tissue-specific manner. In summary, phylogenetic analysis suggests an early origin of spectraplakin-like proteins (Figure 4A,E) [57]. We found the CH domain in combination with GAS2 in Amoebozoa grouped with other CH-GAS2 proteins, along with the human G2L1 protein (Figure 4A). Different CH-GAS2 proteins comprise a well-supported clade in the reconciliation phylogenetic tree. Moreover, these proteins form a distinct group in the phylogenetic tree built based on the CH domain only (Supplementary Figure S4). Actinins, spectrins and spectraplakins form another well-supported group. Both actinins (CH-SR-EF), which are considered as precursors of spectrin-based proteins [57], and CH-GAS2 proteins are found in Amoebozoa. This indicates that these two types of cytoskeletal crosstalk proteins were already present in the common ancestor of Amoebozoa and Opisthokonta (Figure 4E). Spectrins and spectraplakins share a common feature, the SH3 motif, which is not present in actinin, or any of the other analysed proteins. It is likely that the common ancestor of spectrin and spectraplakin containing CH-SR-EF modules acquired SH3 motif before their evolutionary split. This divergence probably occurred during early evolution of opisthokonts and is supported by the presence of both spectrin alpha and beta in Choanoflagellata. Precisely, ancestral spectrin containing all these modules (CH-SR-EF) was ancestor of spectraplakins and later it was split to spectrin alpha and beta. As another line of evidence, we have found the different intermediate forms of CH/SR/GAS2 containing proteins in Fungi (Figure 4A). In a most simple scenario, we speculate that GAS2 could be acquired to the spectraplakin progenitor after divergence from spectrins (Figure 4E) and this places the divergence of the spectraplakin ancestor from the spectrin ancestor at least at the base of Opisthokonta evolution before emergence of Fungi. There are also other possible scenarios; however, based on our phylogenetic analysis they are more unlikely and complex (they involve more events of domain loss and/or gain) and will not be discussed here in more detail.

Taking all this into account, spectraplakins were present at the base of Metazoa evolution, with almost all the basic modules evolved much earlier and arose from primordial actinin. Specific changes in the SH3 motif and the multiplication of the spectrin repeat modules specific to the spectraplakin proteins can be detected in early metazoans, as shown by the presence of the plakin domain in Cnidaria. However, several lines of evidence indicate much earlier emergence of ancestral spectraplakins, at the base of Opisthokonta evolution. Finally, the PR module originated as a hotspot for interaction with IFs in early Bilateria. The specific arrangement of spectraplakin modules suggests the evolution of new, more complicated functions of these proteins, which likely provided a new plethora of interaction partners and ways to regulate cytoskeletal crosstalk in animals.

### 2.4. Dataset Analysis Revealed MISP as a New Player in Human Cytoskeletal Crosstalk

**Figure 5:**
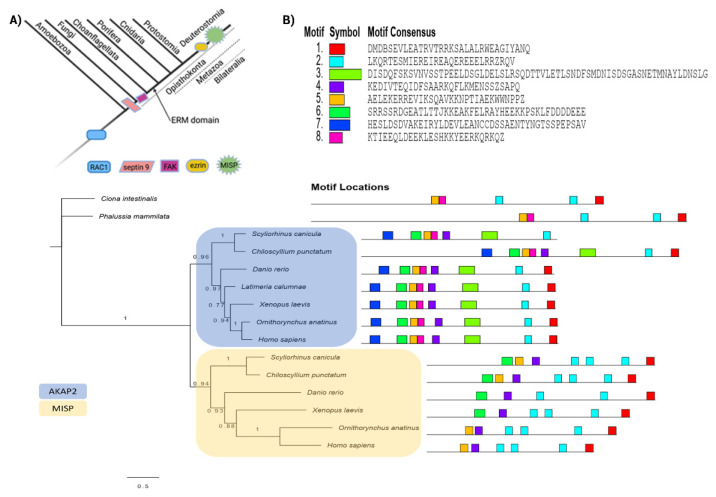
Analysis of evolutionary origins of crosstalk proteins found at intersection of 3 cytoskeletal protein datasets. (**A**) Evolutional emergence of crosstalk proteins found in 3 datasets starting with RAC1 (blue), followed by septin 9 (red), FAK (purple), ezrin (yellow) and MISP (green) as the newest cytoskeletal crosstalk player detected in this analysis. Although ezrin is evolutionary young protein, ERM domain is present before Metazoa. (**B**) Identification of conserved repeated motifs was obtained by MEME suite; motif consensuses are designated in upper right box. Phylogenetic and conserved domains analysis of MISP and its homologs in different Chordata species (lower box). ML tree of MISP homologs, aLRT values are used to infer branch support, values on the main branches are indicated. *Phallusia mammilata* and *Ciona intestinalis* (Chordata, Tunicata) homologs are placed as outgroup. Sequences IDs are listed in Supplementary Table S1 (Sheet 7).

Among obtained proteins found at the intersections, five proteins emerged as players in interaction between focal adhesions, microtubules, and actin filaments: FAK, RAC1, septin 9, MISP, and ezrin. Homologs to some of these proteins were previously found in distantly related organisms. Based on these studies, we conclude a likely evolutionary origin of the specific protein found in the triple intersection in our study (Figure 5A). Rac1 was identified in Amoebozoa [70], and is highly conserved with 91% sequence identity to human RAC1. Direct FAK and septin homologs have been found in unicellular opisthokonts [71]. In addition, septins are known to be involved in many cellular processes in Fungi and Metazoa [72]. In contrast, ezrin appears to be specific to vertebrates. However, ERM family paralogs are highly conserved among themselves (about 75% sequence identity in human ERM proteins) and the ERM domain itself also originates in unicellular opisthokonts [17]. This suggests that early ERM domain proteins likely serve similar functions and have similar interaction partners as human ezrin. Finally, MISP appears to be specific to vertebrates. Previously, MISP was identified as homolog to proteins of AKAP family [73]. In addition, vertebrates possess another MISP homolog, named MISP3. Human MISP and AKAP2 share a conserved C-terminal domain (Figure 5B). However, these proteins are poorly characterized, especially with respect to the binding domains. Interestingly, the human MISP protein is only 62% identical to the mouse and porcine proteins. BLAST hits obtained using human MISP as a search query did not return MISP homolog in Chondrichthyes *R. typus* and *C. milli*, present in our organism dataset. However, when BLAST was performed against all Chondrichthyes, direct MISP homologs were found in two species (*Scyliorhinus canicula* and *Chiloscyllium punctatum*), which was further confirmed by reciprocal BLAST, suggesting that the MISP homolog was present at the base of Vertebrata evolution. Outside Vertebrata, we detected homologs of AKAP2 only in Tunicata species. Distant homologs have been observed previously [73]; however, they have low overall homology with human proteins and generally with MISPs from Vertebrata.

To better understand their evolutionary relationships, we constructed a maximum likelihood tree (ML) with the Vertebrata orthologs of AKAP2 and MISP proteins (Figure 5B). Multiple motifs and repeats are shared by these proteins and are even occasionally found in invertebrate homologs [73]. Because we found MISPs at intersection of different components of the cytoskeleton, we can assume that they have multiple motifs for binding to other proteins or complexes involved in triple crosstalk. However, further experimental studies are required to determine MISP-interacting amino acid residues. In general, the MISP proteins appear to be specific, rapidly evolving proteins of the Vertebrata whose role in the complexes that maintain the triple crosstalk of the cytoskeleton remains undiscovered.

### 2.5. The Evolution of Signal Transduction to Microtubules through CMSC and the KANK-KIF21 Axis Revealed Its Origin in Metazoa

Processes such as the evolution of movement (flagella and cilia in unicellular organisms) and the development of multicellularity, differentiation, and complex movements, as well as the precise segregation of large amounts of genetic information, make the evolution of the cytoskeleton one of the prerequisites for the emergence of highly complex genomes [74]. Therefore, communication between cytoskeletal filaments is one of the key topics for understanding the above functions. Among the proteins linking MTs and FAs, we found many proteins belonging to the cortical microtubule stabilisation complex (CMSC) (Supplementary Table S1, Sheet 8). These complexes are linked to FA via KANK proteins [75]. KANK proteins are important regulators of the crosstalk between MTs and FAs and as such represent good candidates for analysis of the evolution of the MT-FA axis. In addition, previous studies have identified interaction motifs with both KIF21 at MT side and talin at FA side. Both KANK1 and KANK2 can bind talin via the KN motif [75,76] and KIF21A via the ankyrin domain [77]. Structural analysis by Bouchet et al. showed that the KN domain of KANK1 binds to the talin rod domain R7 [76]. A single mutation in talin abolishes the binding of KANK1, suggesting that the interaction between talin-1 and KANK1 plays an important role in the association processes between the outer rim of FAs and MTs. At the C-terminus of the KANK protein, ANK repeats are responsible for binding to KIF21A. KANK1 recruits KIF21A to control MT growth, while on the other hand, KIF21A affects the translocation of KANK1 to the plasma membrane [77]. In addition to its role as a cortical MT growth inhibitor, KIF21A contributes to the formation of overlapping clusters that include PPFIA1 (liprin α1), PPFIBP1 (liprin β1), and PHLDB2 (also known as LL5β) at the cell cortex [78]. MT localization at the cortical site is regulated by plus-end tracking proteins such as CLASPs by forming a complex with ERC1 and PHLDB2, while the N-terminus of liprin β1 has been shown to be responsible for binding to the KANK proteins [79,80].

**Figure 6:**
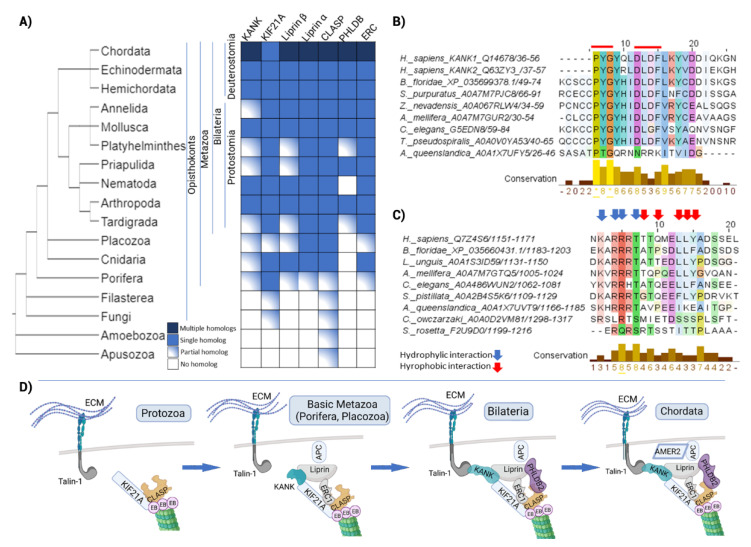
Analysis of the evolutionary origins of CMSC complexes as microtubule-focal adhesion linkers. (**A**) Presence of cortical microtubule stabilisation complex (CMSC) component homologs in different Amorphea groups. The search for homologs was performed as described in M&M, hits obtained after initial BLAST search to human queries were reciprocally BLAST to verify the best hits. A protein was marked as a partial homolog if the coverage was <40% compared to the human protein, while in the case of KIF21A and KANK, conservation of known interaction motifs with KANK and talin was used, respectively. (**B**) Conservation of KN motif in KANK (talin-binding), residues involved in specific interactions with talin are marked with red line. (**C**) Conservation of KBD (KANK-binding domain) in KIF21A, residues involved in specific interactions with KANK are marked with red/blue arrows. (**B**,**C**) 0→* increasing amino acid (aa) residues conservation, * = aa residues in all sequences are conserved. (**D**) Increase in complexity of CMSC components from unicellular opisthokonts to Chordata. Although CMSC originate in the premetazoa, they gradually acquire new components during animal evolution.

Because CMSC (and CMSC-related proteins) is one of the well-described cytoskeletal crosstalk complexes studied to date, we analysed the evolution of CMSC protein components, including interaction of KIF21A as a key player in MT-FA linkage by KANK (Figure 6A, Supplementary Figures S5–S9). The presence of CMSC protein homologs within different Amorphea groups is shown in Figure 6A. The search for homologs was filtered to all species within each analysed group, while NCBI BLAST was performed to check for the presence of homologs in other organisms not included in our dataset. Obtained proteins were also checked by InterProScan. A direct homolog of the plus-end TIP protein CLASP can be found in Amoebozoa, Apusozoa, and unicellular opisthokonts, but the degree of homology is less than 20% (Figure 6A). Phylogenetic analysis (Supplementary Figure S5) reveals divergent CLASP homologs in Protozoa (*T. trahens* and *C. owczarzaki*), early-branching Metazoa, and Protostomia. The CLASP1 and CLASP2 paralogs of fishes (cartilaginous and bony) and humans share a common origin, likely due to CLASP duplication at the base of vertebrate radiation. In humans, the CLASP protein interacts with ERC1 and PHLDB2 through its C-terminal domain [80]. Despite the low overall homology, the C-terminal domain of CLASP is highly conserved in distant organisms that lack ERC1 and PHLDB2 homologs (not shown). It is possible that this region is also important for interaction with some other proteins. In addition, a direct KIF21A homolog was identified in unicellular opisthokonts. As shown in Supplementary Figure S6, the homolog from *T. trahens* branches with human KIF4A, indicating that the origin of KIF21A is related to the evolution of Opisthokonta. Overall, the CMSC complexes as found in humans, did not evolve before the emergence of Metazoa (Figure 6A), contributing to the evolution of key Metazoan features, such as multicellularity, movement, and tissue specialization.

Direct homologs of ERC1, liprin and KANK, can also be identified in Porifera. All of these proteins are present as single homologs in early-branching Metazoa, but with less than 40% coverage to human proteins, indicating the absence of some domains (Figure 6A, Supplementary Figures S7 and S8) [81]. Human KANK and liprin interact through their N-terminal regions, while the N-terminus of ERC1 is linked to the liprin CC2 region (aa 259–542) [79,82]. These domains in CMSC homologs of Porifera are at least partially conserved. However, because no precise interacting motifs/residues are reported, we cannot discuss whether these interactions might be established in these simple animals. The last examined CMSC protein, PHLDB2, has origins in Bilateria. According to our phylogenetic analysis, the closest homolog from Cnidaria species *S. pystillata* is more closely related to human KIF16B protein (Supplementary Figure S9). Thus, nearly all CMSC components are present in all Bilateria, including APC protein, suggesting that these complexes were completed before the divergence of Deuterostomia and Protostomia.

In a further step, we analyse in more detail the direct MT-FA axis via the KANK proteins. KANK is known to bind to talin via the KN motif and to KIF21A via ANK repeats, providing a direct link between MT and FA [76,77,83]. The major residues involved in talin binding are PYG and LDLF patches within the KN motif [84]. Interestingly, despite the presence of KANK homologs, we did not detect a conserved KN motif in Porifera and other non-Bilateria animals (Figure 6A,B). In addition to running BLAST, we also scanned the KANK homologs using InterProScan to search for KN motifs. We also searched our local database using KN motif seed and Leitmotif server. Of the 28 Metazoa (excluding Craniata), we found the KN motif in the KANK protein in three of four Mollusca, two Nematoda, three of six Arthropoda (all Insecta), and all four non-Craniata deuterostomes proteins. It is likely that this motif originated in the basal Bilateria but has been lost in some lineages. In any case, “KANK” homologs lacking the KN motif probably cannot bind talin in the manner described in the literature, resulting in their inability to connect FAs to MTs. On the other hand, the R7R8 domain of talin is mostly conserved (not shown) to the most distant organisms in our data set, indicating the importance of this region as a common binding hub.

We also analysed the other side of this axis, the linkage between KIF21 and KANK. KIF21A homologs are present in all groups studied except Amoebozoa and Apusozoa (Figure 6A, Supplementary Figure S6). The structure of KIF21A with KANK via KANK1-binding peptide domain (KBD) was resolved, showing that two short α-helices in the KIF21A KBD peptide occupy the groove formed by the capping domain and the first ANK repeat of KANK1 [83]. Although KIF21A homologs can be found in Fungi and unicellular opisthokonts, its binding partner KANK is absent. In Porifera, the KIF21A homolog has a partially conserved KBD-like domain, with residues involved in the hydrophilic interaction with the ANK repeat more conserved than amino acids involved in the hydrophobic interaction (Figure 6C). Alignment of the ankyrin domain and comparison of the KIF21A binding residues in KANK also show a high degree of conservation (Supplementary Figure S10). This suggests a likely interaction between these two proteins in basic Metazoa. However, further in-depth modelling and experimental analyses are needed to determine whether the KANK protein from sponges can bind to KIF21A.

In Cnidaria, the KBD site is almost the same as that of the human KIF21A protein (Figure 6C), as well as the interacting residues in the ankyrin domain of KANK (Supplementary Figure S10), probably allowing mutual interaction. Finally, using CMSC as an example, we have shown the gradual evolution of these cellular structures (Figure 6D), which increase in complexity with the addition of new proteins and protein domains, and can be traced from unicellular opisthokonts to complex animal forms. The interaction between MTs and FAs via KANK or spectraplakins likely originated in the earliest animals. However, given the number of proteins we have found at the interface of MTs and FAs, the other proteins and complexes that perform crosstalk between these components of the cytoskeleton should also be considered [85,86,87]. The nature and precise role of these interactions remain to be characterized experimentally.

## 3. Conclusions

The aim of this study was to summarize the proteins important for cytoskeletal crosstalk in humans and to explore the evolutionary background of the proteins found at the intersections of the cytoskeletal datasets. The spectraplakin protein dystonin emerged from the analysis as the only protein found in all datasets examined, consistent with previous work asserting the role of spectraplakin proteins in cytoskeletal crosstalk [27,28]. Based on our analysis, it appears that complete spectraplakin proteins, comprising the necessary domains for interaction with all components of the cytoskeleton, arose first in Bilateria (Figure 4E). However, the various spectraplakin modules came together much earlier, at the onset of the evolution of Opisthokonta, and were able to communicate between microtubules and actin filaments in these simple life forms. In comparison, the origins of vertical crosstalk by CMSC complexes are associated with metazoan emergence (Figure 6D). However, considering the number of highly conserved homologs mediating crosstalk between FAs and actin filaments or microtubules, which have not been studied in detail here, it is probable that other vertical crosstalk mechanisms have a much earlier origin. In summary, specific crosslinker domains or multiprotein complexes that contribute to cytoskeletal crosstalk in animals have gradually increased in complexity from the base of metazoan evolution, consistent with the evolution of more complex cell and tissue complexes.

**Figure 7:**
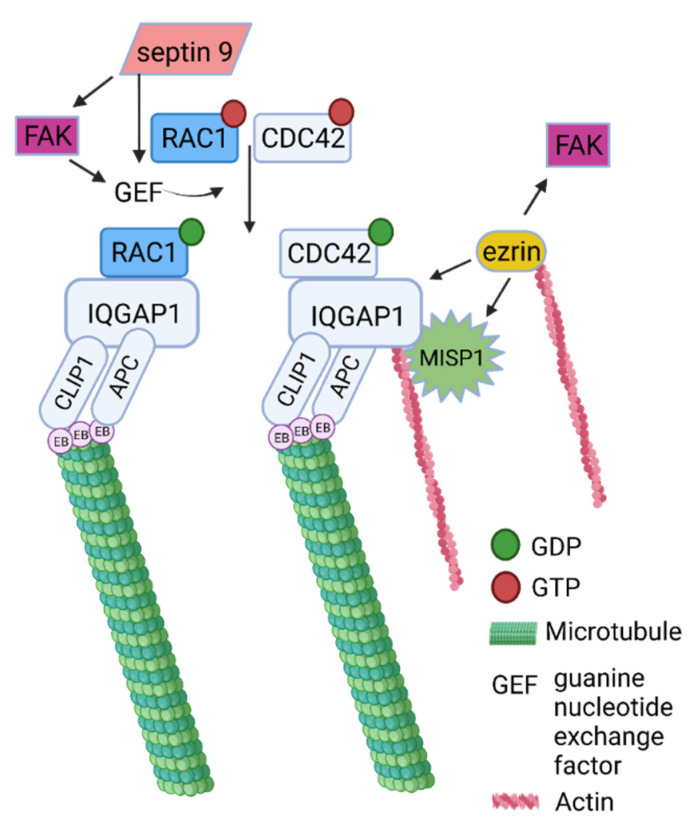
Suggested pathway involving five proteins found at the intersections of FA, MT and actin filament datasets. The hypothetical mechanism involves RAC1, septin 9, FAK, ezrin and MISP depicted together with common interacting partners.

Finally, five proteins emerged as possible players in crosstalk between FAs, MTs and actin filaments: FAK, RAC1, ezrin, septin 9, and MISP. Here we propose the mechanism that includes all proteins found at the intersections of three datasets (Figure 7). FAK has been known to play the main role in cytoskeleton remodelling, participating in many processes including the regulation of Rho-family GTPases (CDC42, and RAC1) [36]. Another member of cytoskeletal proteins with GTP-ase activity, septin 9 has been shown to regulate cytoskeletal processes through FAK/Src/paxillin signalling pathway [71]. In addition, knockdown of septin 9 decreases the levels of CDC42, RhoA, and RAC, which are important members in the activation of IQGAP, a scaffold protein found at the intersection of FAs and MTs that plays a key role in regulating cell motility processes. Ezrin, another protein detected at the intersections of FAs, MTs, and actin filaments, directly binds IQGAP and affects its cortical localization [88]. In addition, Vodicska et al. showed that MISP interacts directly with terminal part of IQGAP, enabling the binding of MISP to CDC42 through IQGAP [89]. They also showed that MISP knockdown promotes cortical accumulation of IQGAP, and IQGAP mediated CDC42 deactivation. IQGAP is responsible for the capturing of the astral MTs to the cell cortex, in collaboration with CLIP1 (also known as Clip-170) or APC. Activated CDC42 and RAC1 are key regulators of cell polarization that orchestrate the crosstalk between actin and MTs by activating IQGAP, through formation of a ternary complex with APC. When bound together, this complex possesses the ability to bind CLIP1, attaching to the plus-end of the microtubules via its interaction with EB proteins [90,91,92].

In summary, we have shown the importance of the evolutionary studies of the cytoskeletal crosstalk to elucidate the development of complex interfilamentary mechanisms and interactions that likely contributed to the development of motility and tissue specialization. However, this study represents the first insight into the evolution of general cytoskeleton crosstalk. Further bioinformatic and experimental studies are needed to uncover signal transduction through cytoskeletal filament associations with focal adhesions.

## 4. Methods

### 4.1. Protein Dataset Assessment—Updating a List of Proteins Belonging to Human Cytoskeleton

The list of proteins linking integrin-related adhesion complexes (IACs) to intermediate filaments, microtubules, and the actin cytoskeleton is described in Supplementary Table S1 (Sheets 1–4). The preliminary dataset containing the list of proteins (Supplementary Table S1) was defined using the online gene ontology tool QuickGO [93]. When using an interactive search tool for genes and gene products, the following conditions were set: (a) taxon: *Homo sapiens* (9606); (b) gene products: proteins; (c) references: any Pubmed; (d) evidence: experimental evidence used in manual assertion (ECO:0000269), including its Child terms. Furthermore, the following GO terms were used: “actin cytoskeleton (GO:0015629)”, “microtubule cytoskeleton (GO:0015630)”, “intermediate filament cytoskeleton (GO:0045111)”, and “focal adhesion (GO:0005925)”. QuickGO database was assessed in November 2021 to retrieve human cytoskeletal proteins. Furthermore, obtained datasets were curated according to the published datasets. Briefly, the Intermediate filament dataset is expanded with the proteins from the Human Intermediate Filament database [94]. The microtubule dataset is enriched by proteins curated in the literature [10,95,96]. In addition to the QuickGO dataset, the FA list is defined by adding the proteins from the literature curated dataset [97] and the consensus adhesome proteins [98]. In addition, recent datasets obtained on long-term cultured cells were compared with the meta-adhesome [98], while the matched proteins were added to the focal adhesion dataset [97,98,99,100]. Overall, the dataset contains the proteins responsible for integrin-mediated signalling and focal adhesions to microtubules, actin filaments, and intermediate filaments. The intersections of the generated protein datasets were performed using the Venn diagram online tool Venny (2.1.0 software version) [101].

### 4.2. Clustering of Human Cytoskeleton Proteins

Cluster Analysis of Sequences (CLANS) has been used to identify groups of proteins within the human cytoskeleton. CLANS is a Java utility based on the Fruchterman-Reingold graph layout algorithm [102]. It runs BLAST on given sequences, all against all, and clusters them in a 3D space according to their similarity. Thus, all retrieved proteins are clustered into groups based on sequence similarities. A 2D representation was obtained by randomly distributing sequences in an arbitrary distance space. Groups were determined using the in-tool option network clustering (10, true, true) (Supplementary Table S1, Sheet 5).

### 4.3. Model Organism Dataset

To investigate the evolutionary development of cytoskeletal crosslinking complexes, taxa belonging to the supergroup Amorphea (formerly known as Unikonts) were included in the organism dataset. These include Metazoa (animals) and their closest relatives: fungi and protists, which also belong to Opisthokonta clade, and Apusozoa and Amoebozoa as the closest sister groups [103]. Furthermore, NCBI taxonomy was used as the basis for species relationships [104]. Species were selected using datasets from previous studies [105,106]. In the final dataset, metazoan taxa were represented with at least 1 species for each class; several protists (1 Choanoflagellatea, 1 Filasterea, 1 Ichthyosporea, and Corallochytrium), 3 fungal species, *T. trahens* (Apusozoa), and *Dyctyostelium discoideum* (Amoebozoa) (Supplementary Table S1, Sheet 9). When necessary for the construction of the phylogenetic trees, protein sequences from additional organisms are added to the dataset. The taxonomic structure describing the final dataset is shown in Supplementary Table S1 (Sheet 9) and Supplementary Figure S2, and is based on the topologies used by the NCBI Taxonomy database [107] and the Tree of Life project [108,109].

### 4.4. Data Collection

Local BLAST proteome databases were created using the Blast2GO tool, version 5.2.5 [110]. This database, created from representative proteomes, was then used to search for protein homologs, using human proteins as queries. For the simplification of the analysis only canonical isoforms were used in this study. The following parameters were used in the analysis: Blast expectation value less than 1 × 10^−5^, High scoring segment pair length cutoff = 33. Positive hits were matched to the human database at BLAST to determine if the obtained protein was the best match to the human query protein (Supplementary Table S1, Sheet 8). Hierarchical clustering (Euclidean distance; Linkage method: average) between proteins within each cut group was performed separately using Morpheus software [111].

### 4.5. Identification and Analysis of Protein Conserved Domains

Proteins were scanned using InterProScan, version 5.55–88.0, within InterPro to determine functional domains [112,113]. HMMER, version 3.3.2 [114], or Leitmotif server, version 2.0 [115] were used to search for “hidden homologs” that were not detected using BLAST. For this purpose of Leitmotif scan, seed motifs were built based on multiple sequence alignments (MSAs), conserved domains previously described in the literature, and InterProScan results. These motifs were then used to search our local proteome database using default parameters. Hits with scores > 0 were considered significant. SMART version 9 [116] and CDD search, version 3.19 [117] were used for further domain architecture analysis. Motif enrichment analysis was performed with MEME Suite [118].

### 4.6. Phylogenetic Analysis

Multiple sequence alignments were created using the MAFFT tool, version 7 [119]. Jalview, version: 2.11.2.0 w used for MSA analysis and manual editing [120]. Additionally, Gblocks server was used for the removal of variable regions within MSA [121]. All MSA are available upon request.

Phylogenetic trees were constructed using the maximum likelihood method in PhyML 3.0 [122]. aLRT values (approximate likelihood ratio test) were used to infer branch support. The aLRT values above 0.7 were indicated on the phylogenetic trees. For the phylogenetic analysis of spectraplakins (Figure 4A) four domains (CH, GAS2, SR1 and last SR in actinins, spectrins, spectraplakins, and fungal SR-GAS2 proteins) were separately aligned using MAFFT or ClustalW implemented in Geneious^®^ 9.1.8 and concatenated into one alignment [123]. Maximum-likelihood (ML) phylogenetic analysis was performed using iqtree version 2.0 [124]. The best-fit model for each individual alignment was selected using ModelFinder [125] and the branch supports were assessed with ultrafast bootstrap approximation [126] and SH-like approximate likelihood ratio test [122].

FigTree software, version 1.4.4, was used for statistical analysis and graphical representation of the results. PhyloT, version 2, was used to create phylogenetic trees of the species [127].

## Data Availability

All data generated or analyzed during this study are included in this published article and its Supplementary Information Files.

## Supplementary Materials

The following supporting information can be downloaded at: https://www.mdpi.com/article/10.3390/ijms23105594/s1.
