# Supplementary material for: Ancient Origins of Cytoskeletal Crosstalk: Spectraplakin-like Proteins Precede the Emergence of Cortical Microtubule Stabilization Complexes as Crosslinkers
Source: Int J Mol Sci. 2022 May 17;23(10):5594. doi: 10.3390/ijms23105594 (PMC9145010; doi:10.3390/ijms23105594)
Supplement: Supplementary file 1 [file ijms-23-05594-s001.zip › Supplementary Figures.pdf]

Supplementary Materials

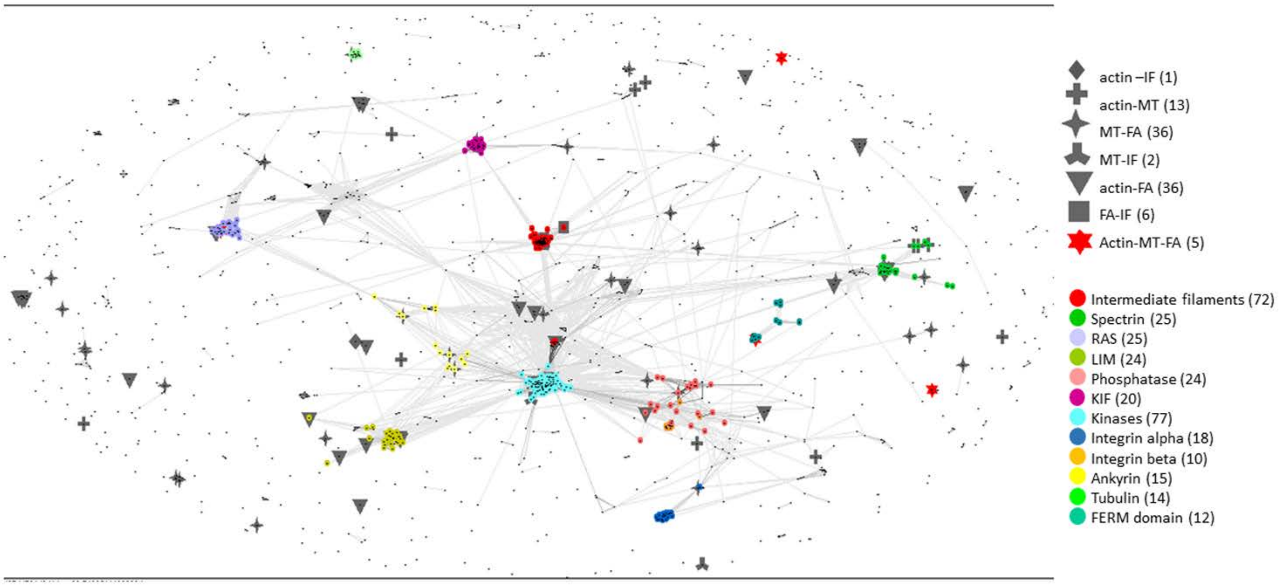

Figure S1. CLANS clustering of human cytoskeleton proteins. Different protein families are assigned with different colors, while intersection proteins are depicted with different symbols.

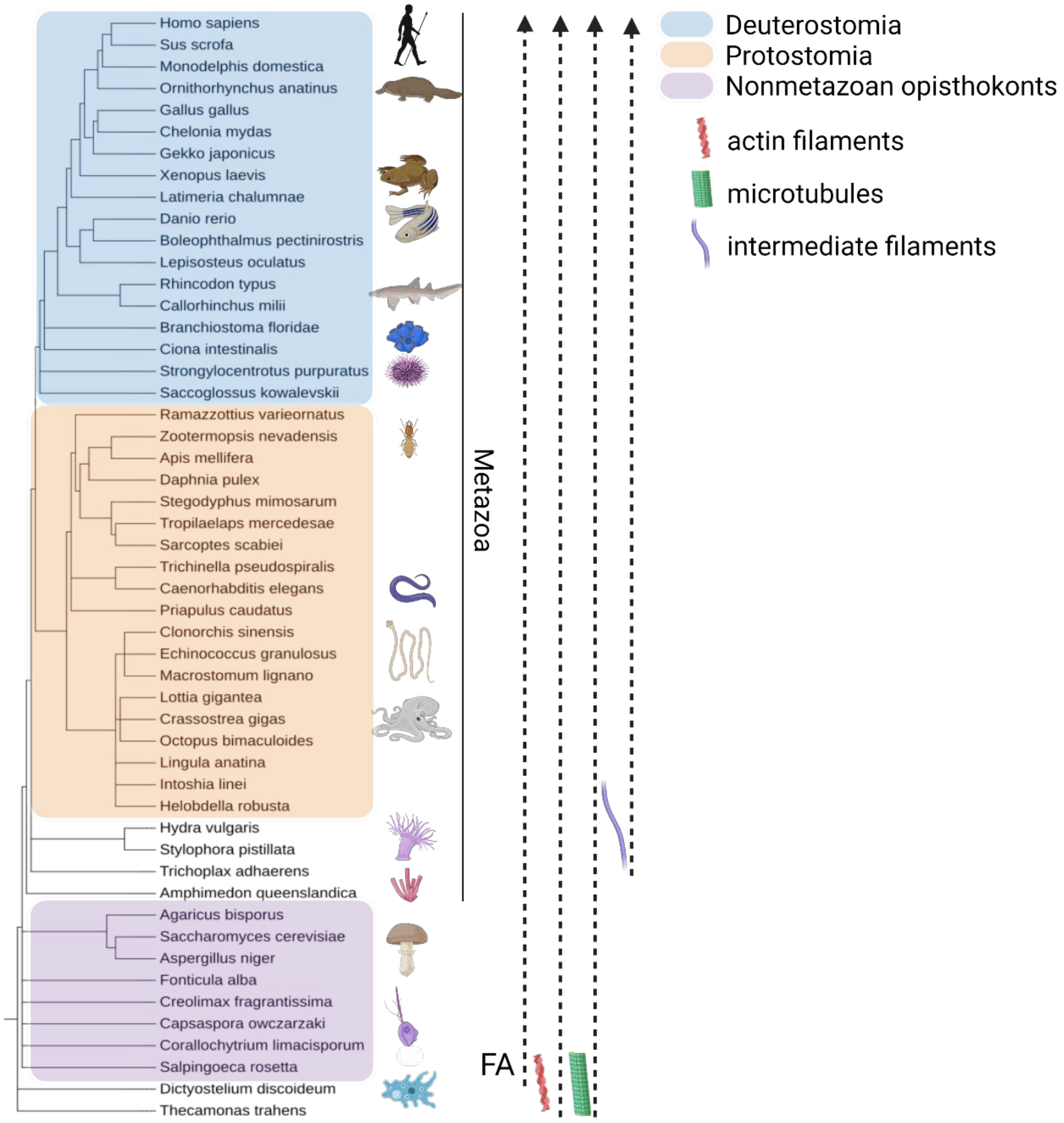

Figure S2. Phylogenetic tree made by PhyloT tool containing organismal dataset used in this study. Presence of different cytoskeletal components in these organisms is designated on the right.

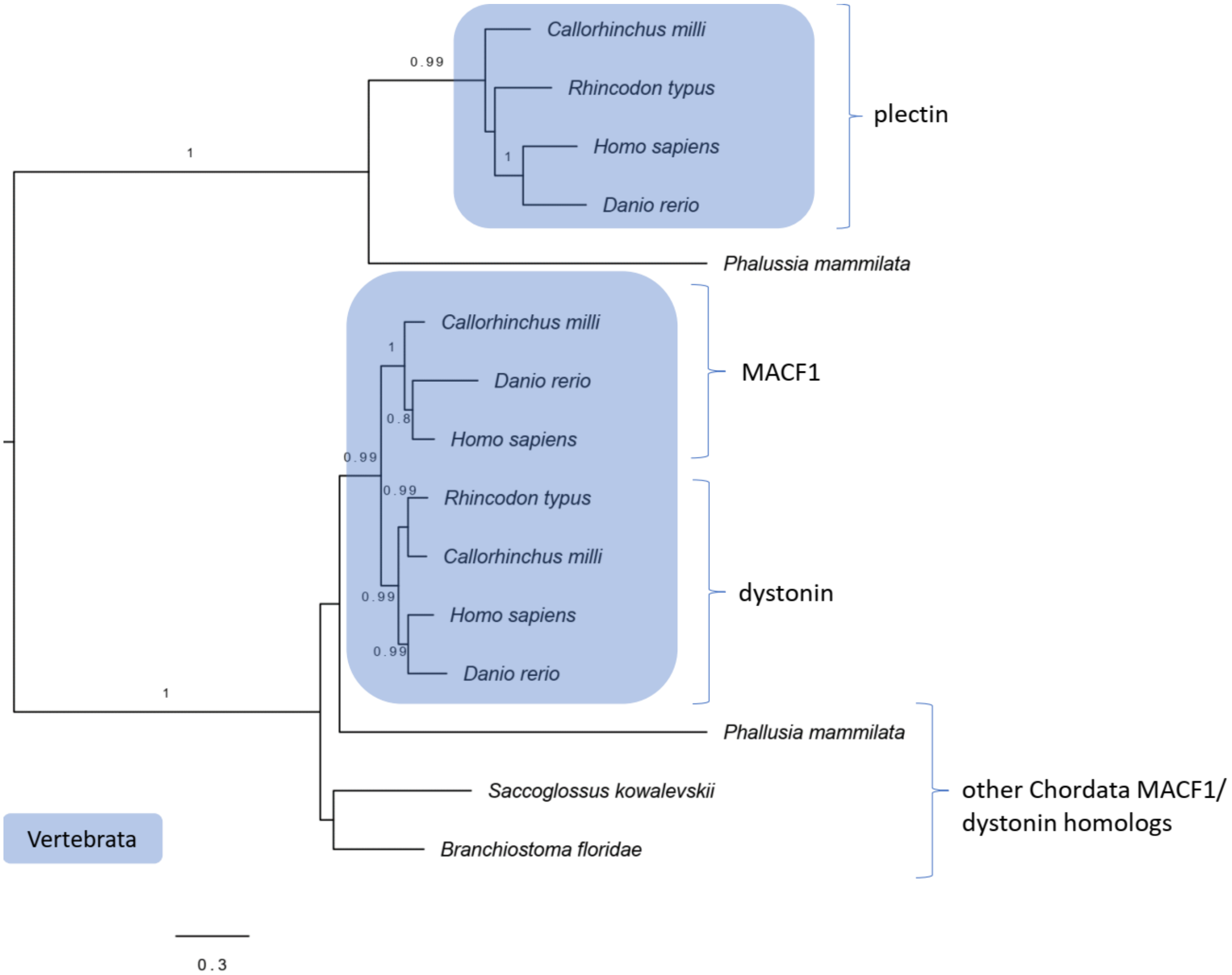

Figure S3. ML tree of Chordata spectraplakins (dystonin and MACF1) and plakin (plectin) proteins. The best-fit model according to AIC criterion was LG+G+T. aLRT values > 0.7 are shown inferring branch support. Sequences IDs are listed in Supplementary Table 1.

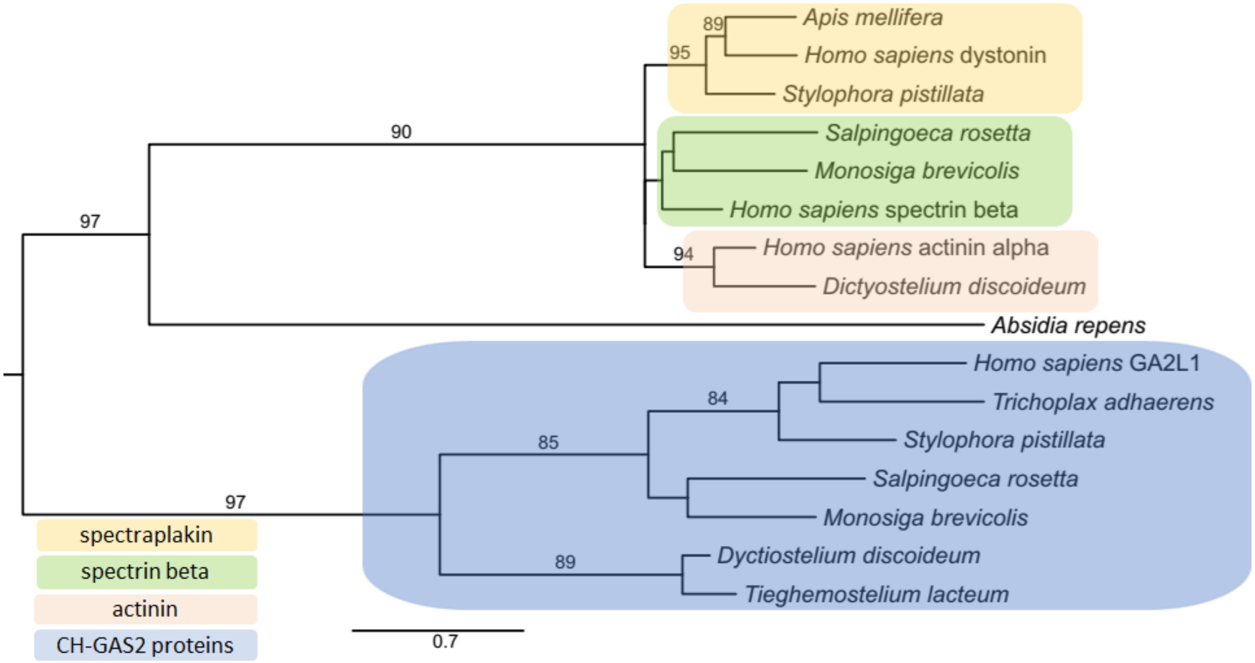

Figure S4. Phylogenetic relationship of CH domains that are present in different spectrins, spectraplakins and GAS2 proteins. The phylogenetic tree was reconstructed based on MSA of the CH domain using a maximum likelihood method. The best-fit model according to BIC criterion was LG+G4. Branch support is presented with UFBoot values (1000 replicates). Only >80 values are shown. The tree is midpoint rooted. IDs of sequences used to built the tree are indicated in the Supplementary Table 1.

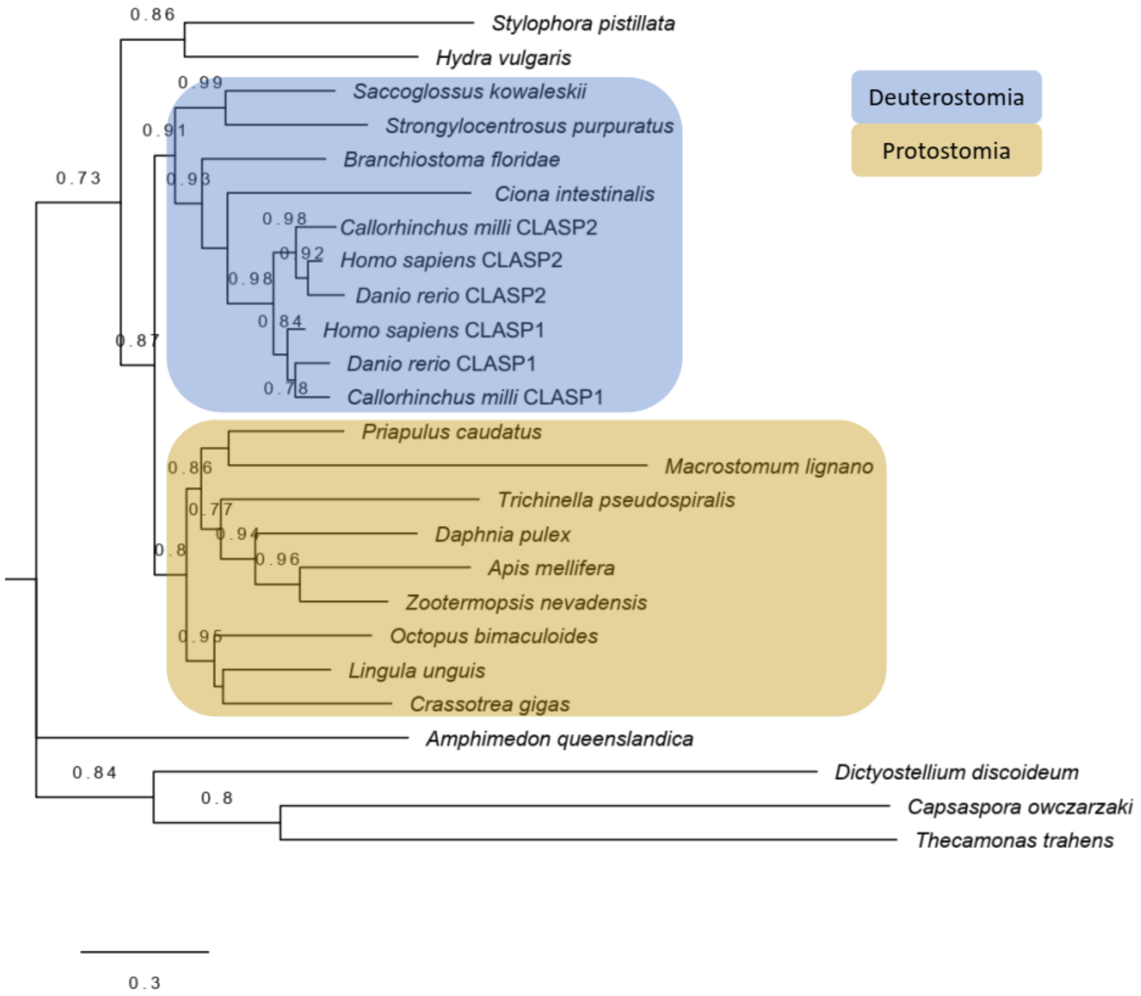

Figure S5. ML tree of CLASP homologs in Opisthokonta and Apusomonada.

The best-fit model according to AIC criterion was LG+G+I+F. aLRT values > 0.7 are shown to infer branch support. IDs of sequences used to build the tree: **Ddi** Q55BD8, **Aqu** A0A1X7V8T9, **C.o.** A0A0D2WMS0, **Tth** A0A0L0DWT2. **Mli** A0A267FQM7, **Hvu** XP_012553551.1, **Spi** A0A2B4SA59, **Zne** A0A067R2S2, **Dpu** E9HJY5, **Ame** A0A7M7M181, **Tps** A0A0V0XXH6, **Pca** XP_014663049, **Cgi** K1QCK4, **Lun** A0A1S3HCG9, **Obi** A0A0L8GA74, **Cin** F6W6K9, **Bfl** XP_035658510.1, **Spu** A0A7M7N026, **Sko** XP_006818149, **Hsa** CLASP1 Q7Z460, CLASP2 O75122, **Cmi** CLASP1 A0A4W3IJN8, CLASP2 A0A4W3JU88, **Dre** CLASP1 F1R253, CLASP2 F1R161.

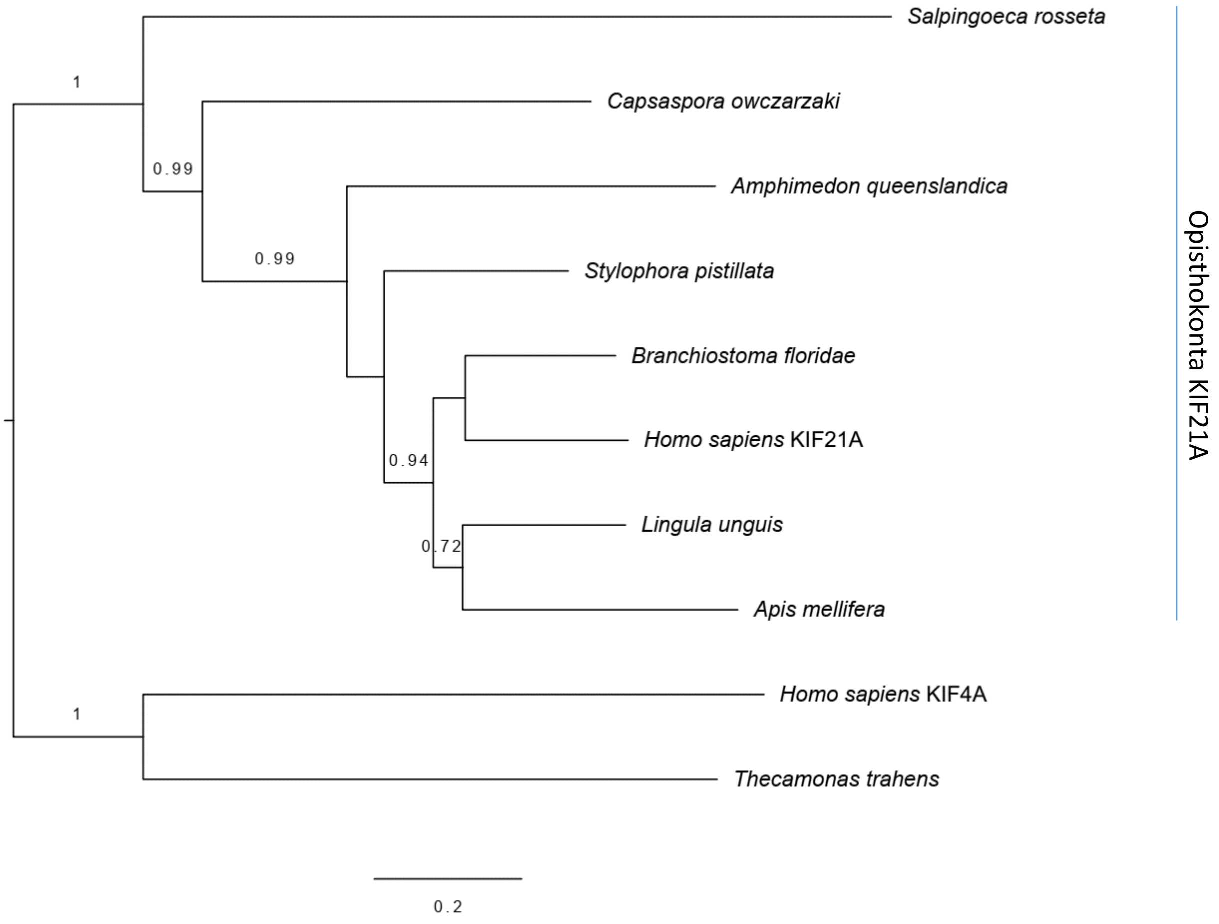

Figure S6. ML tree of KIF21A homologs in Opisthokonta. The best-fit model according to AIC criterion was LG+G+I+F. Closest KIF21A homolog from *T.trahens* (Apusomonada) is branching with human KIF4A. aLRT values > 0.7 are shown to infer branch support. IDs of sequences used to build the tree: **Hsa** KIF4A O95239, KIF21A Q7Z4S6, **Tth** A0A0L0DLG6**, Cow** A0A0D2VM81**, Sro** F2U9D0**, Aqu** A0A1X7UVT9**, Spi** A0A2B4S5K6**, Ame** A0A7M7GPK1**, Lun.** XP_013396200.1**, Bfl** XP_035660431.1.

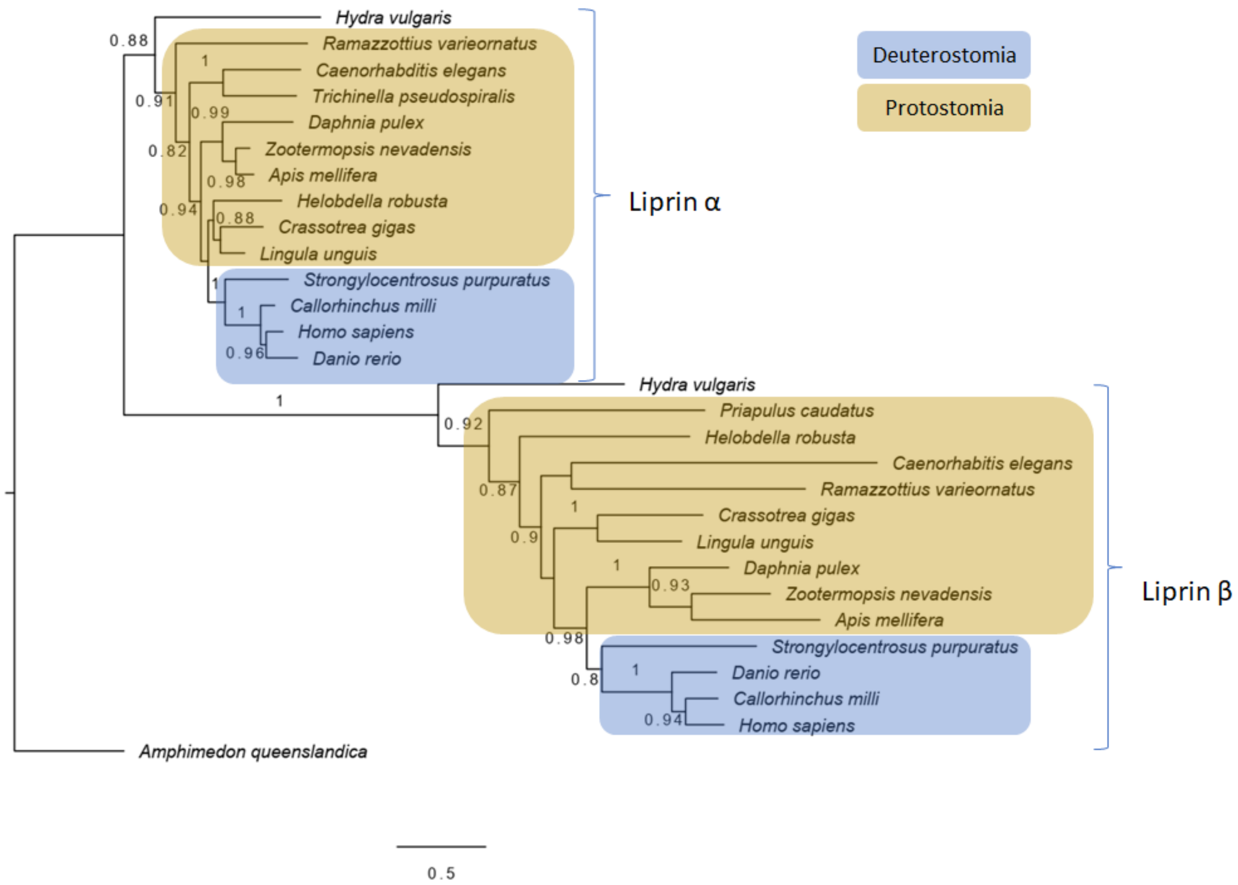

Figure S7. ML tree of liprin homologs in Metazoa. Eumetazoa species have both liprin α and liprin β homologs, while *A.queenslandica* (Porifera) has single predicted liprin protein.
The best-fit model according to AIC criterion was LG+G+I+F. aLRT values > 0.7 are shown to infer branch support. IDs of sequences used to build the tree: **Aqu** A0A1X7V6S1; **liprin** ***α***: **Hvu** XP_004206607.1, **Rva** A0A1D1W4G4, **Cel** Q21049, **Tps** A0A0V0YF80, **Hro** T1FRP9, **Cgi** K1R1P0, **Lun** A0A1S3KA15, **Ame** A0A7M7MU22, **Dpu** E9FRQ5, **Zne** A0A067RU54, **Spu** A0A7M7P265, **Hsa** Q13136, **Dre** A9JR75, **Cmi** A0A4W3JHY5; **liprin β**: **Hvu** XP_012556355.1, **Pca** XP_014673509.1, **Hro** T1EGH9, **Rva** A0A1D1V8S0, **Cel** Q94071, **Cgi** K1PZJ9, **Lun** A0A1S3JF91, **Zne** A0A067REV5, **Dpu** E9GYB0, **Ame** A0A7M7L1J8, **Spu** A0A7M7P464, **Hsa** Q86W92, **Dre** B7ZVC4, **Cmi** A0A4W3IRG3.

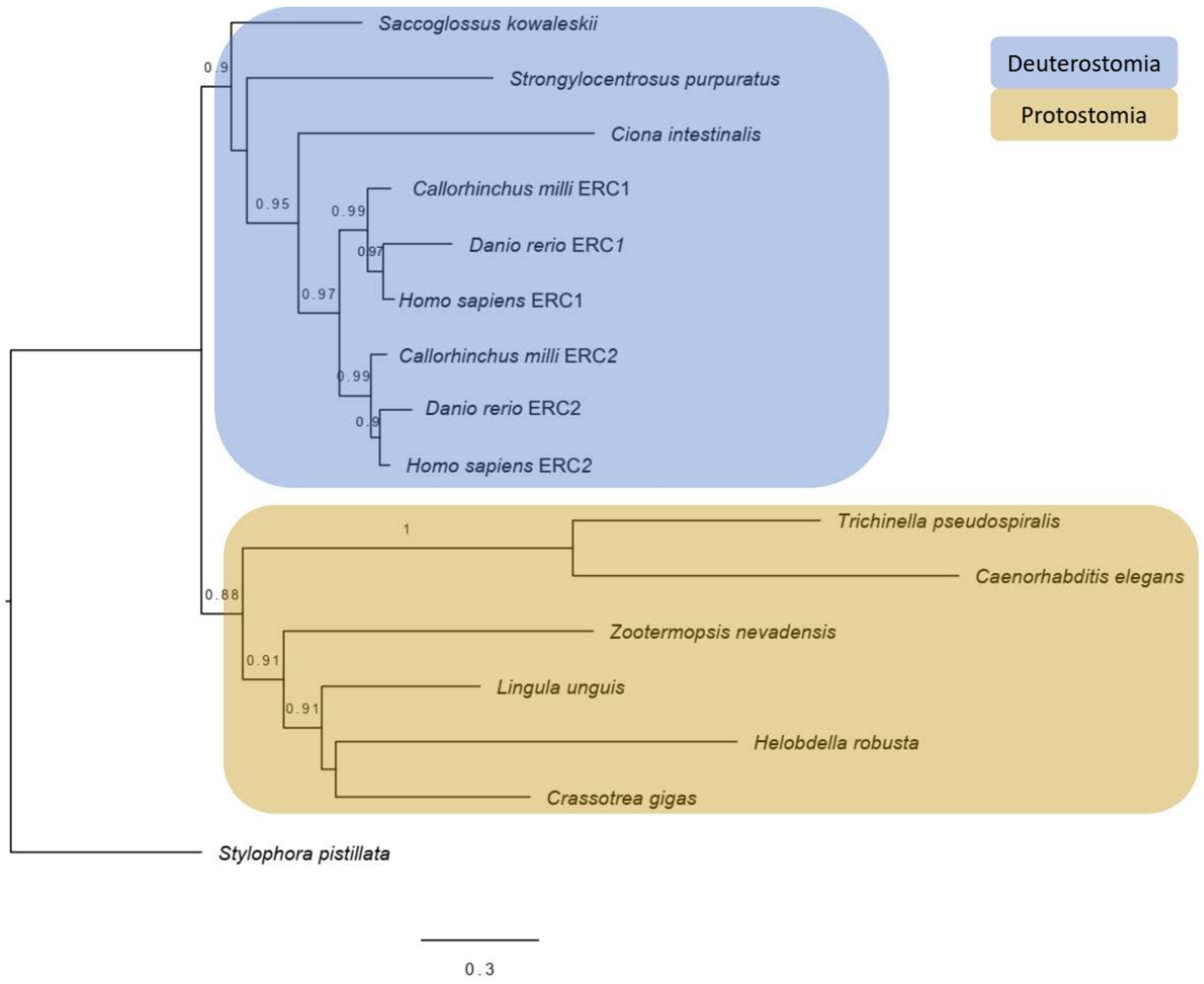

Figure S8. ML tree of ERC homologs in Metazoa. The best-fit model according to AIC criterion was JTT+G+I+F. aLRT values > 0.7 are shown to infer branch support. IDs of sequences used to build the tree: **Spi** A0A2B4SSH7, **Tps** A0A0V0XLN2, **Cel** O44490, **Cin** F6SDZ0, **Cmi** ERC1 A0A4W3K904, ERC2 A0A4W3GYW2, **Dre** ERC1 B7ZVS4, ELKS2 A0A0R4IGY4, **Hsa** ERC1 Q8IUD2, ERC2 O15083, **Spu** A0A7M7MX96, **Sko** XP_006817071.1, **Hro** T1FWH3, **Lun** A0A1S3HA77, **Cgi** K1QCT1, **Pca** XP_014676835.1, **Rva** A0A1D1UTD1, **Ame** A0A7M7LQU9, **Zne** A0A067QWS7.

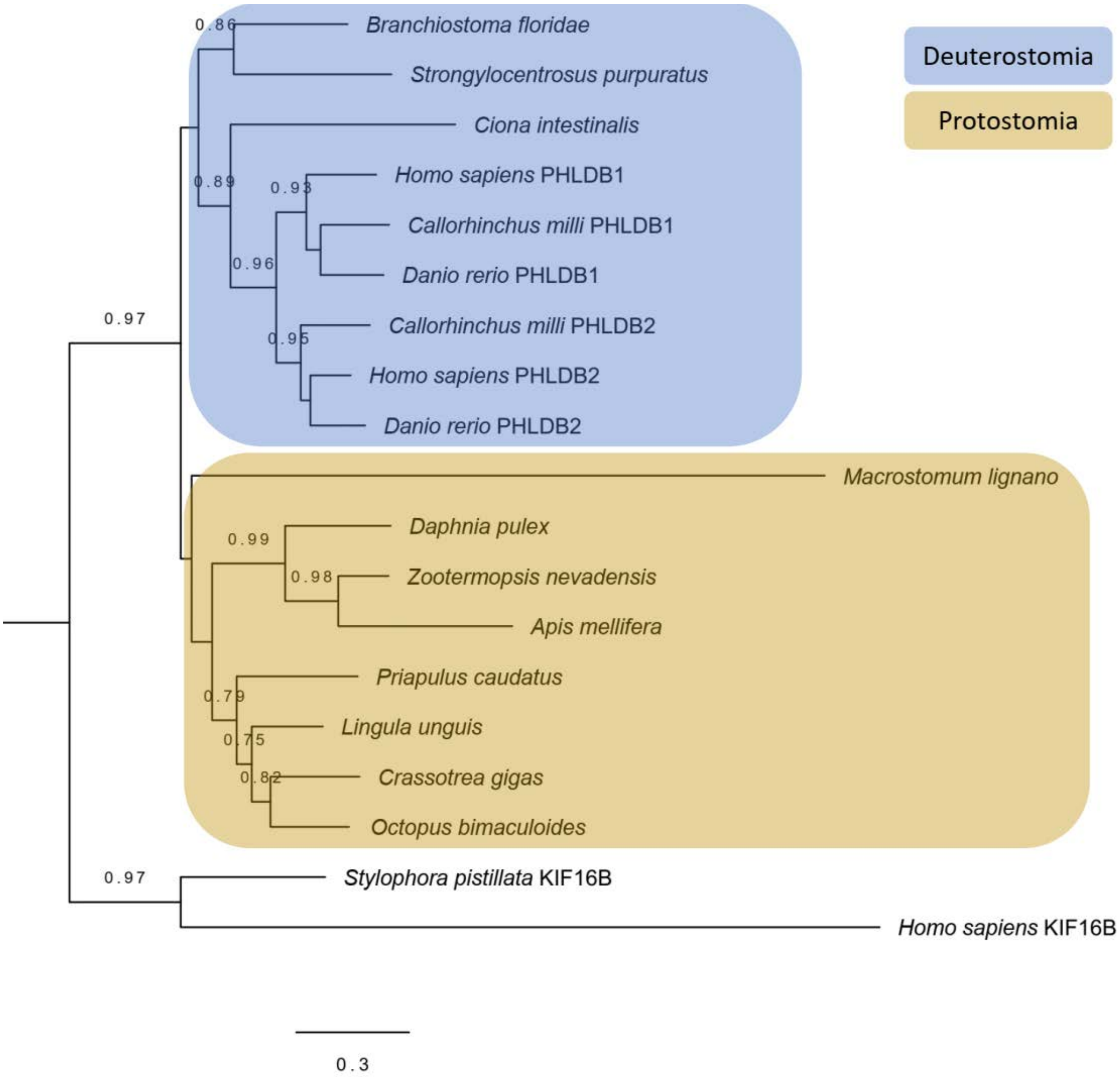

Figure S9. ML tree of PHLDB homologs in Bilateria. PHLDB homolog from *S.pistillata* (Cnidaria) is more closely related to *H.sapiens* KIF16B. The best-fit model according to AIC criterion was LG+G. aLRT values > 0.7 are shown to infer branch support. IDs of sequences used to build the tree: **Hsa** PHLDB2 Q86SQ0, PHLDB1 Q86UU1, KIF16B Q96L93, **Cmi** PHLDB1 A0A4W3GZF7, PHLDB2 UPI001C3F7021, **Dre.** PHLDB1 A2BFP0, PHLDB2 A0A0R4IA44, **Cin** F6PV66, **Bfl** C3XRU4, **Spu** A0A7M7NB47, **Dpu** E9G6U6, **Ame** A0A7M7L0H7, **Zne** A0A067QIX4, **Mli.** A0A267GFC2, **Lun** A0A1S3JKI4, **Pca** XP_014665519, **Obi** UPI00071CB49E, **Cgi** K1RLW8, **Spi** A0A2B4SY28.

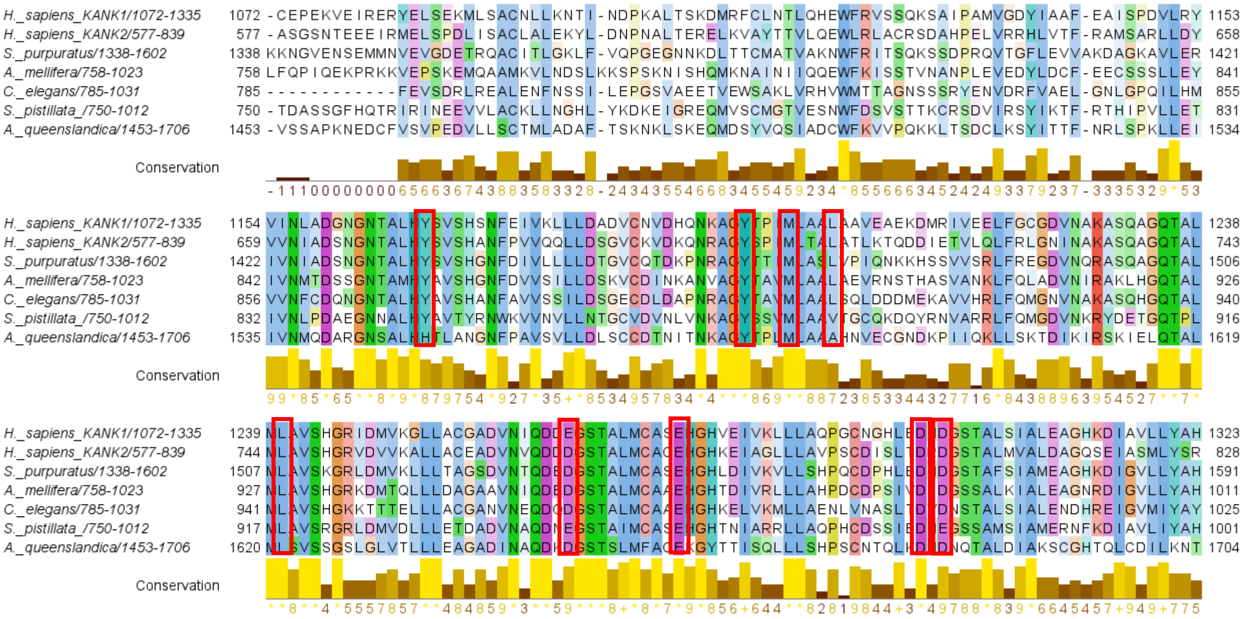

Figure S10. Multiple sequence alignment of ankyrin domain of KANK proteins from different animals. KIF21A binding residues are indicated by red boxes. Overall amino acid residues conservation is indicated below alignment; 0→* increasing conservation. IDs of sequences used for the alignment: **Hsa** KANK1 Q14678, KANK2 Q63ZY3, **Spu** A0A7M7PJC8, **Ame** A0A7M7GUR2, **Cel** G5EDN8, **Spy** A0A2B4S5K6, **Aqu** A0A1X7UFY5.
